# Molecular Phylogeny of the Family Cordulegastridae (Odonata) Worldwide

**DOI:** 10.3390/insects15080622

**Published:** 2024-08-19

**Authors:** Thomas Schneider, Andy Vierstraete, Oleg E. Kosterin, Dietmar Ikemeyer, Fang-Shuo Hu, Rodolfo Novelo-Gutiérrez, Tom Kompier, Larry Everett, Ole Müller, Henri J. Dumont

**Affiliations:** 1 Independent Reseacher, Arnold-Knoblauch-Ring 76, 14109 Wannsee, Germany; 2 Ehrenamtlicher Mitarbeiter Museum für Naturkunde, 10115 Berlin, Germany; 3 Department of Biology, University of Gent, 9000 Gent, Belgium; andy.vierstraete@ugent.be (A.V.); henri.dumont@ugent.be (H.J.D.); 4 Institute of Cytology & Genetics SB RAS, Academician Lavrentyev Avenue 10, 630090 Novosibirsk, Russia; kosterin@bionet.nsc.ru; 5 Independent Researcher, Billerbecker Str. 6, 48329 Havixbeck, Germany; dkjikemeyer@t-online.de; 6 Dragonfly Association of Taiwan, 2F., No. 5, Long’an Rd., Xinzhuang Dist., New Taipei City 242046, Taiwan; fangshuo_hu@smail.nchu.edu.tw; 7 Natural History Museum of Denmark, University of Copenhagen, Zoological Museum, 1350 Copenhagen, Denmark; 8 Instituto de Ecología A.C, Red de Biodiversidad y Sistemática, Carretera Antigua a Coatepec 351, El Haya, Xalapa 91070, Veracruz, Mexico; rodolfo.novelo@inecol.mx; 9 Independent Researcher, Schoutenstraat 69, 2596 SK Den Haag, The Netherlands; kompierintokyo@yahoo.com; 10 Independent Researcher, 1902 Cedardale Lane, Knoxville, TN 37932, USA; fattigia@aol.com; 11 Independent Researcher, Birkenweg 6d, 15306 Libbenichen, Germany; mueller.ole@gmail.com

**Keywords:** *Anotogaster*, *Cordulegaster*, *Thecagaster*, *Neallogaster*, *Zoraena*, synonyms

## Abstract

**Simple Summary:**

Cordulegastridae, recognized for their striking black-and-yellow colouring, are robust and large dragonflies typically found in clean running springs and streams, which are unfortunately facing significant threats worldwide. Currently, 52 species are listed in this family. Cordulegastridae are remarkably uniform in the structure of the male appendages and the female valvular scale, while the pattern of yellow markings on the abdomen and thorax may vary even within a taxon. This often results in confusion regarding the identification, distribution, and intraspecific division of many species in this family. To address these challenges, we undertook a molecular phylogenetic analysis of this family. Our analyses supported most of the traditional genera. The well-known *bidentata* group of the current *Cordulegaster*, including *C*. *coronata* and its sister species *C. brevistigma*, was transferred by us to the genus *Thecagaster*. The genus *Neallogaster* remained unresolved. However, *Cordulegaster pekinensis*, currently known as *Neallogaster pekinensis*, was placed by us in the genus *Thecagaster* as well. The genus *Zoraena* stat. rev. was recovered to include most of the American members of Cordulegastridae, except for *C. virginiae* and, tentatively, *C. diadema*, which were retained in the genus *Cordulegaster* along with the members of the *Cordulegaster boltonii* group. The monophyly of the genus *Anotogaster* was confirmed, and three dubious species of this genus were synonymized. Our revision provides a clearer understanding of the evolutionary relationships and taxonomic framework of the family Cordulegastridae.

**Abstract:**

In this study, we present the first attempt at a molecular phylogenetic analysis of the entire family of Cordulegastridae involving 60% of its known species. Our analysis is in favor of reclassification of the members of the family into four genera: (i) the monophyletic genus *Anotogaster* Selys, 1854, with the number of known species reduced by three synonymizations; (ii) the genus *Cordulegaster* Leach in Brewster, 1815 including all members of the *boltonii* group and, as a preliminary solution, the American species *C. virginiae* Novelo-Gutiérrez, 2018 and, very tentatively, *C. diadema* Selys, 1868. The *bidentata* group forms a genus of its own, for which we restored the name *Thecagaster* Selys 1854, stat. rev. *Cordulegaster pekinensis* McLachlan in Selys, 1886, currently considered as *Neallogaster pekinensis,* was placed by us in *Thecagaster* as well. The genus *Neallogaster* Cowley, 1934 needs further investigation involving all remaining species listed in it. The genus *Zoraena* Kirby, 1890, stat. rev., was recovered to accommodate the remaining American species of *Cordulegaster*. We synonymized three species of *Anotogaster*: *Anotogaster gregoryi* Fraser, 1923 = *Anotogaster xanthoptera* Lohmann, 1993, syn. nov.; *Anotogaster* *kuchenbeiseri* (Förster, 1899) = *Anotogaster antehumeralis* Lohmann, 1993, syn. nov.; *Anotogaster kuchenbeiseri* (Förster, 1899) = *Anotogaster cornutifrons* Lohmann, 1993, syn. nov., based on examination of the existing type specimens. The type of specimens of *A. klossi* Fraser, 1919 = *A. flaveola* Lohmann 1993 syn. confirm., were also examined, and their synonymy was confirmed. The isolated populations of *A. sieboldii* (Selys, 1854) from the archipelagos of Okinawa and Amami Oshima in Japan, respectively, should be regarded as separate species, which will be described elsewhere. Furthermore, we suggest the synonymization of *Cordulegaster parvistigma* Selys 1873 syn. nov. with *Thecagaster brevistigma* (Selys 1854) comb. restaur.

## 1. Introduction

Cordulegastridae is a Holarctic family that is relatively poor in species, 52 of which are currently listed [1]. They are rheophilic insects inhabiting trickles, brooks, and rivulets. Members of this family are characterized by large size, robust build, and black and yellow colouration. Cordulegastridae are remarkably uniform in the structure of their male appendages and the female valvular scale, while the pattern of yellow markings on the abdomen, thorax, and occipital triangle may vary within even a single taxon, thus challenging species identification (for examples, see [2,3,4,5]). This has resulted in repeated confusion regarding the identification, distribution, and intraspecific division of many species in this family. 

According to the present taxonomic concept [1], the family consists of three genera: *Cordulegaster* Leach in Brewester, 1815, *Anotogaster* Selys, 1854, and *Neallogaster* Cowley, 1934. The genus *Cordulegaster* occurs in North Africa, Europe, and South-West Asia, extending eastwards as far as China and Japan [6,7,8]. Additionally, there is a North American group of *Cordulegaster*, with two species extending into Central America [9,10]. The genus *Neallogaster* occurs in Asia from the Hindukush over the Himalayan Mountains to China. Its species are confined to higher altitudes (>2000 m a.s.l.); only *Neallogaster pekinensis* (McLachlan in Selys, 1886) [11] occurs at lower altitudes, between 500 and 1500 m a.s.l. There were doubts on the placement of *Cordulegaster pekinensis* in the genus *Neallogaster* by Lohmann [12] because it lacks the specific features of the frons [13], and we deny it (see below). The genus *Anotogaster* is restricted to East Asia, with most species recorded from China and Vietnam. The western boundary of this genus is located in Northeastern Pakistan and Kashmir. 

A recent comprehensive revision of *Cordulegaster* of Western Palaearctic has clarified the taxonomic status of the species occurring across North Africa, Europe, the Middle East, and the western part of Central Asia [5,14]. However, a taxonomic revision of the entire family was still missed.

The research history of this family includes some of the earliest works on dragonflies. The species most familiar to Europeans, currently known as *Cordulegaster boltonii* (Donovan, 1807), started to be repeatedly described as long ago as the 18th century under many no longer valid names, including *Libellula grandis* Scopoli, 1753, *L. forcipata* Harris, 1780, *Aeshna annulata* Latreille, 1805, etc. The genus *Cordulegaster* Leach in Brewster, 1815, based on this species, was described 62 years after the first of the above-mentioned available but invalid specific names were proposed [15]. *Cordulegaster insignis* Schneider, 1845, was described based on a female from Kellemisch (Turkey) [16]. One year later, Kolenati [17] described a further species from the Caucasus, *Aeshna charpentieri,* presently *Cordulegaster charpentieri* (Kolenati, 1846). The latter taxon had a complicated history [5]. *Cordulegaster coronata* Morton, 1916, was described from the Ferghana Valley in Central Asia [18] and ranges in the eastern part of the Western Palaearctic from North-East Iran to Kyrgyzstan and probably to North-West China. This taxon was later treated as a subspecies of *C. insignis* [5,7,19], but recently, the species status of *C. coronata* was confirmed by molecular phylogenetic analysis [5]. In the same study, two new species were recognized, and several others were synonymized. Even in such extensively studied regions as North and Central America, new species have been discovered recently, for example, *C. sarracenia* Abbott & Hibbitz, 2011 from North America [20] and *C. virginiae* Novelo-Gutiérrez, 2018 from Mexico [10]. 

Selys [21] divided the species currently attributed to Cordulegastridae into two genera: *Cordulegaster,* with seven species, and a new genus, *Thecaphora* Selys, 1854, proposed for *T. diastatops* Selys, 1954. He also split *Cordulegaster* into four subgenera, of which three were described as new: *Anotogaster* Selys, 1854 (for two species, *A. basalis* Selys, 1854 and *A. nipalensis* Selys, 1854), *Thecagaster* Selys, 1854 (for *T. brevistigma* Selys, 1854), and *Taeniogaster* Selys, 1854 (for *Aeshna obliqua* Say, 1840). It should be noted that Selys used the names that he claimed to be subgeneric for binominal combinations of species as if they were generic, e.g., *Anotogaster basalis*. Selys [21]. He did not indicate thetype species of *Anotogaster*, so its type species, *A. nipalensis*, was subsequently designated by Kirby [22]. Later, Selys [23] added the genus *Allogaster* Selys, 1878 (for *A. latifrons* Selys, 1878). However, at present, only two of these five genera/subgenera (*Cordulegaster* and *Anotogaster*) are accepted unequivocally, and two of them (*Thecaphora* and *Allogaster*) have changed their names. 

Kirby [22] proposed the replacement of the generic name *Thecaphora*, which appeared a junior homonym, with a new name, *Zoraena* Kirby, 1890. Later, Carle [24] supported this genus and associated with it his new species *Z. bilineata* Carle, 1983. Later, Lohmann [12] even established a new subfamily, Zoraeninae, for this genus. However, conflicting opinions persisted, with several studies rejecting the genus *Zoraena*, along with its subfamily [9,25,26,27,28]. Tennessen [29] and Abbott and Hibbitts [20] did not accept the genus *Zoraena* but grouped the North American representatives of *Cordulgaster* into two groups: (i) the *diastatops* group, corresponding to the genus *Zoraena* including also *C. bilineata*, *C. sarracenia*, *C. sayi* Selys, 1854 and *C. talaria* Tennessen, 2004, and (ii) an unnamed group containing the remaining of American *Cordulegaster* species. Later, Tennessen [29] restored *Zoraena* as a valid genus based on larval morphology.

The name *Allogaster* Selys, 1878, appeared to be a junior homonym of a genus of Coleoptera. Hence, Cowley [30] replaced it with *Neallogaster* Cowley, 1934 for this formal reason. The difference between *Neallogaster* and *Cordulegaster* is not clear, and the taxonomy of Indian and Nepalese species in comparison with those from China is not clarified. *Neallogaster* currently contains nine species [1]. Selys [23] characterized this genus (as the subgenus *Allogaster*) by the extraordinary expansion of the frons. Asahina [31] tentatively accepted both genera *Neallogaster* and *Cordulegaster* but pointed out that “it seems rather difficult to separate the two genera without surveying whole the representatives of both the genera”. This difficulty was also emphasized by van Pelt [2], who argued that the separation of *Neallogaster* and *Cordulegaster* is impossible without surveying all representatives of both genera. Several species that are now allocated to *Neallogaster* were previously considered for *Cordulegaster* [2,3] and the exact placement of the different species in this genus remains still unclear.

The genus *Anotogaster* was separated from other genera of Cordulegastridae by the two male characteristics: the absence of the anal triangle in the hind wings and the absence of the auricles, both present in other genera ([21] and subsequent authors). Currently, 14 species of *Anotogaster* are accepted [1]; most of them are large to giant insects. They all exhibit a striking similarity in the general habitus and colour pattern, so their confusion is easy, while it is nearly impossible to separate species by comparing females. As a consequence, several doubtful species were described, especially those based solely on one female, for example, *A. xanthoptera* Lohmann, 1993 [32] and *A. flaveola* Lohmann, 1993 [32]. Additionally, two other *Anotogaster* species described by Lohmann [32], *A. antehumeralis* Lohmann, 1993 [32] from the northern slope of the Kunlun Mountains and *A. cornutifrons* Lohmann, 1993 [32] from Central China, have never been reported since their original description, so their taxonomic status remained enigmatic.

A few more available generic names have been proposed in Cordulegastridae, but they are not currently considered valid. The genus *Sonjagaster* Lohmann, 1992 was proposed by Lohmann [12] for *C. insignis*; later, he included more species in this genus. The reception of this genus was equivocal, and it was synonymized with *Cordulegaster* in the recent revision of the West Palearctic species [5]. Lohmann [12] described four more genera for American species: *Archegaster* Lohmann, 1992 (type species *Cordulegaster sayi* Selys, 1854), *Kalyptogaster* Lohmann, 1992 (type species *Cordulegaster erronea* Hagen, 1878), *Lauragaster* Lohmann, 1992 (type species *Cordulegaster diadema* Selys, 1868), and *Pangaeagaster* Lohmann, 1992 (type species *Cordulegaster maculatus* Selys, 1854), none of which were accepted by odonatologists. Finally, Yousuf & Yunus [33] described the genus *Kuldanagaster* Yousuf & Yunus, 1974 for their *Kuldanagaster pakistanica* Yousuf & Yunus, 1974, which was later revealed to be a synonym of *Cordulegaster brevistigma* (Selys, 1854) [34].

### Aim of the Study

Many taxonomic problems in Cordulegastridae on the generic and species levels still remain unresolved. To unravel them, we undertook a molecular phylogenetic investigation using two well-established markers: the nuclear ITS region (the internal transcribed spacer of the ribosomal RNA gene cluster, further in the text referred to as ITS for simplicity) and the barcoding fragment of the mitochondrial COI gene (cytochrome oxidase I, further on referred to as COI). The aim of this study was to elucidate the evolutionary relationships in Cordulegastridae and, hence, to suggest a justified taxonomic classification of the family. This phylogenetic inference was then used to approve or synonymize the existing species. For this purpose, we searched for the type specimens of dubious species in museums and, when available, studied their morphology. By integrating molecular phylogenetics with morphological methods, we aimed to provide a robust framework for classifying Cordulegastridae, thereby contributing to a better understanding of their biodiversity and evolutionary history.

## 2. Materials and Methods

Our molecular analysis included a total of 281 Cordulegastridae specimens. Of them, 43 specimens were sequenced in the course of this study (Table 1). A total of 77 specimens were sequenced and published by us previously [5,35], and the sequences of 163 specimens were obtained from GenBank [36]. Six sequences that were left unidentified in GenBank were also involved, their species attribution being inferred from our analyses (Table 2). Our analysis involved specimens of all currently recognized genera of Cordulegastridae and 34 of the 52 species currently listed in this family [1].

**Table 1:** Information on Cordulegastridae specimens newly sequenced for this study.

Species	Country	Locality	Collector/Reference	DNA No.	GenBank COI	GenBank ITS
***Anotogaster*** Selys, 1854						
*A. chaoi* Zhou, 1998	Vietnam	Sa Pa, Lao Cai	Kompier, T. leg.	676	PP792819	PP806539
*A. chaoi*	Vietnam	Sa Pa, Lai Chau	Kompier, T. leg.	677	PP792820	PP806540
*A. chaoi*	Vietnam	Sa Pa, Lao Cai	Kompier, T. leg.	678	PP792821	PP806537
*A. gigantica* Fraser, 1924	Vietnam	Yen Bai	Kompier, T. leg.	685	PP792828	PP806544
*A. klossi* Fraser, 1919	Vietnam	Axan, Tay Giang	local collector, coll. Schneider, T.	591	PP792839	PP806546
*A. klossi*	China	Hunan, near Huaihua	local collector, coll. Schneider, T.	592	PP792838	PP806545
*A. klossi*	Vietnam	Pia Oac, Cao Bang	Kompier, T. leg.	682	PP792841	no data
*A. klossi*	Vietnam	Bach Ma, Thua Thien–Hue	Kompier, T. leg.	683	PP792840	PP795698
*A. myosa* Needham, 1930	China	Qin Ling Mountains, Shaanxi	team Siniaev, V. leg. coll. Schneider T.	671	PP792824	PQ120580
*A. nipalensis* (Selys, 1854)	Nepal	Shivapuri Mountains, 2100 m	Brockhaus, T. leg.	451	PP792823	PP806543
*A. sakaii* Zhou, 1988	Vietnam	Pia Oac, Cao Bang	Kompier, T. leg.	679	PP792825	PP806541
*A. sakaii*	Vietnam	Pia Oac, Cao Bang	Kompier, T. leg.	680	PP792826	no data
*A. sakaii*	Vietnam	Tam Dao, Vinh Phuc	Kompier, T. leg.	681	PP792827	PP806542
*A. sapaensis* Karube, 2012	Vietnam	Sa Pa, Lao Cai	Kompier, T. leg.	686	PP792822	PP806538
***Cordulegaster*** Leach in Brewster, 1815						
*C. bidentata* Selys, 1843	Austria	District Melk	Schneider, T. catch and release.	433	PP792852	no data
*C. boltonii* (Donovan, 1807)	Russia	Kostroma Province, Poksha River at Burtasovo village	Kosterin, O. E. & Onishko, V. V. leg.	637	PP792829	PP795700
*C. boltonii*	Austria	District Melk	Schneider, T. catch and release.	428	no data	PP806554
*C. boltonii*	Austria	District Melk	Schneider, T. catch and release.	426	no data	PP806556
*C. boltonii*	Austria	District Melk	Schneider, T. catch and release.	425	no data	PP806555
*C. brevistigma*	Pakistan	Hindukush, North Chitral	Schneider, T. leg.	788	PQ117792	PQ120579
*C. charpentieri* (Kolenati, 1846)	Russia	Dagestan Sergokala District, 4 km SW Myurego village	Kosterin, O. E. & Onishko, V.V. leg.	635	PP792851	PP806547
*C. coronata* Morton, 1916	Afghanistan	Paghman Mountains, near Kabul	Pliushch, I. leg., coll. Schneider T.	572	PP792847	no data
*C. coronata*	Kyrgyzstan	Kyrgyzstan, South of Gyulcha	Ustjuzhanin, P. leg.	636	PP792850	PQ120578
*C. coronata*	Tajikistan	near Sangtuda	Bergmann, A. leg.	672	PP792848	no data
*C. coronata*	Tajikistan	near Sangtuda	Bergmann, A. leg.	674	PP792849	no data
*C. coronata*	Kazakhstan	NE Almaty/ SE Kazakhstan	Nicolai, B. leg., engine grill of a bus.	787	PQ117793	PQ120577
*C. diastatops* (Selys, 1854)	USA	Pennsylvania, Spring Creek near Bank Run Rd., Alleghany NF	Wolbert, J.R. leg.	771	PP792842	PP806552
*C. dorsalis* Hagen in Selys, 1858	USA	Tulare, California Sherman Pass	Rogers, R. leg. coll. Schneider, T.	600	PP792854	PP806548
*C. erronea* Selys, 1878	USA	Tennessee, Hickory Creek, trib., Flanagan Branch, N of Yarnell Rd.	Everett, Jr., L.E. leg.	769	PP792846	no data
*C. erronea*	USA	Tennessee, Holston River trib., off Clark Rd.	Everett, Jr., L.E. leg.	766	PP792845	PP806550
*C. heros* Theischinger, 1979	North Macedonia	Plachkovica	Kitanova, D. leg.	462	PP792830	no data
*C. heros*	North Macedonia	Plachkovica	Kitanova, D. leg.	467	PP792831	no data
*C. heros*	North Macedonia	Maleshevski	Kitanova, D. leg.	481	PP792833	no data
*C. heros*	North Macedonia	Ograzden	Kitanova, D. leg.	465	PP792832	no data
*C. heros*	North Macedonia	Novachani, Veles	Kitanova, D. leg.	478	PP792835	no data
*C. heros*	North Macedonia	Maleshevski	Kitanova, D. leg.	474	PP792834	no data
*C. heros*	North Macedonia	Novachani, Veles	Kitanova, D. leg.	463	PP792836	no data
*C. heros*	North Macedonia	Maleshevski	Kitanova, D. leg.	466	PP792837	no data
*C. maculata* Selys, 1854	USA	Tennessee, Little creek, 46 m below Polecat Hollow in Rugby SNA	Everett, Jr., L.E. leg.	768	PP792844	no data
*C. maculata*	USA	Tennessee, Lyon creek trib., Carter Paschal Park	Everett, Jr., L.E. leg.	767	no data	PP806553
*C. obliqua* (Say, 1840)	USA	Tennessee, Hurricane creek trib. N of Turnpike Rd.	Everett, Jr., L.E. leg.	770	PP792843	PP806551
*C. virginiae* Novelo-Gutiérrez, 2018	Mexico	Veracruz near Xalapa, Sanuario del Bosque de Niebla, 1336 m	Novelo-Gutiérrez, R. leg.	668	PP792853	PP795699
*C. virginiae*	Mexico	Veracruz near Xalapa, Sanuario del Bosque de Niebla, 1336 m	Novelo-Gutiérrez, R. leg.	776	PP792855	PP806549

**Table 2:** Unidentified sequences from GenBank [36] and their proposed identification.

GenBank	GenBank COI	GenBank ITS	Country	Proposed Name
*Anotogaster* sp.	AB708839	AB706945	China; Zhejiang	*Anotogaster myosa*
*Anotogaster* sp.	LC366733	LC366139	Laos	*Anotogaster gregoryi*
*Anotogaster* sp.	AB708840	AB706946	Vietnam	*Anotogaster gigantica*
*Anotogaster* sp.	AB708842	AB706948	Vietnam	*Anotogaster gigantica*
*Anotogaster* sp.	AB708843	AB706949	Vietnam	*Anotogaster gigantica*
*Anotogaster* sp.	AB708845	AB706951	Vietnam	*Anotogaster chaoi*
*Anotogaster* sp.	671 *	failed	China, Shaanxi	*Anotogaster myosa*

* DNA No. of a specimen analyzed in the course of this study (see Table 1).

### 2.1. Phenotype Comparison and Examination of the Types

The name-bearing types of four doubtful species of *Anotogaster* described by Lohmann [32] were searched for as indicated in the original publications:

*Anotogaster xanthoptera*, holotype female (no further specimen mentioned), from Birma (Burma, Myanmar), without any further details of region or capture date, Museum, Koenig, Bonn.

*Anotogaster flaveola*, holotype female, from Taiwan, 23°01′ N, 120°14′ E, V. Rolle leg., Museum für Naturkunde, Berlin.

*Anotogaster antehumoralis*, holotype male, from Xinjiang Province, West China, northern slope of Kunlun Mountains, 1930 m, 36°10′ N, 81°29′ E, 8–10 June 1890, S. Conradt leg., Museum für Naturkunde, Berlin.

*Anotogaster cornutifrons*, holotype male, from Shaanxi Province, Central China, 3 June 1936, E. Suenson leg, Naturalis (Rijksmuseum van Natuurlijke Historie, Leiden Netherlands), Leiden; Paratypes males, the same data as above, female 2 June 1936, the same data as above.

If the type was found, it was compared with the literature; if the typea was lost, the original description was compared with other published literature.

### 2.2. Molecular Procedures

DNA extraction, PCR, and sequencing followed the protocols described in Schneider et al. [5].

Partial sequences of the cytochrome c oxidase subunit I (COI) from mitochondrial DNA and the ITS region (ITS1, 5.8S, and ITS2) from the nuclear DNA were used for the phylogenetic analyses. The sequences of the COI gene fragment are 568 bp long (except for some sequences from GenBank [36] that are only 408 bp long). The ITS region sequences are between 731 and 837 bp long, depending on the species. 

#### 2.2.1. Molecular Phylogenetic Analysis

We sequenced 43 specimens (for which 39 COI and/or 27 ITS sequencings were successful) (Table 1) and used 106 sequences for ITS and 234 for COI, including those from our previous study [5], from GenBank. Accession numbers of the sequences available from GenBank are provided next to the names in the phylogenetic trees presented. In total, our study involved 275 COI sequences and 132 ITS sequences of Cordulegastridae. We used *Aeshna grandis* (Linnaeus, 1758) (Aeshnidae) as the outgroup but also added *Chlorogomphus shanicus* Wilson, 2002 (Chlorogomphidae) to the ITS analysis and *C. shanicus* and *Neopetalia punctata* (Hagen in Selys, 1854) (Neopetaliidae) to the COI analysis to see their position in the trees with respect to Cordulegastridae.

The online version of MAFFT (Multiple Alignment with Fast Fourier Transform) [37] was used to perform the multiple sequence alignments, with the default parameters (Strategy: Auto; Align unrelated segments: Try to align gappy regions anyway; Scoring matrix for nucleotide sequences: 200 PAM/K = 2; Score of N in nucleotide data: nzero; Guide tree: Default).

We determined the model of DNA evolution with JMODELTEST version 2.1.10 [38], using the default parameters (Model Filtering: off; Number of substitutions schemes: 11; Base frequencies: on; Rate variation: Invariable sites and Gamma: on, nCat = 4; Base tree for likelihood calculations: ML optimized; base tree search: NNI (Nearest neighbor interchange)). The best model was chosen based on the Bayesian information criteria (BIC).

The phylogenetic reconstructions were performed using MRBAYES 3.2.7 [39], with the model parameters from JMODELTEST. For both the COI and ITS, the General Time Reversible (GTR) model was used with gamma-distributed rates across sites. For the COI, the invertebrate mitochondrial code was used, and for the ITS, the universal code was used. The MCMC chain was executed for 10 million generations for 2 independent analyses with 4 chains per analysis. Sampling the Markov chains was performed every 1000 generations and 50% of the samples were discarded when calculating the convergence diagnostics. Both phylogenetic trees reconstructed by MRBAYES and shown in this paper reached convergence: for the COI tree, the ESS was 1778 for the combined runs; for the ITS tree, the ESS was 5047 for the combined runs. 

We also reconstructed phylogenetic trees from the same data with the Maximum Likelihood method with IQ-TREE 2.3.5 [40]. We let it estimate the best model of evolution before performing a 50000 ultra-fast bootstrap tree construction with a minimum branch support of 0.7. For the COI, the model was a transitional model (TIM2) with empirical base frequencies and the FreeRate model as Rate heterogeneity across sites. For the ITS, the model was Hasegawa–Kishino–Yano (HKY) with empirical base frequencies, allowing a proportion of invariable sites and FreeRate model as Rate heterogeneity across sites.

A multi-individual multi-locus species tree was constructed in StarBeast [41], using Bayesian coalescent analysis, as implemented in the BEAST package [42,43]. This was applied to both genes. Input files were created in BEAUTI v2.7.6 with the StarBeast3 v1.1.8 template using the HKY + G + I model for both markers. The following settings were used for all analyses: base frequencies ‘empirical’ clock model ‘Strickt clock Clock.rate = 1′; tree prior ‘default values (Yule Model)’. The analyses were run in BEAST v.2.7.6 for 400 million generations, with sampling every 5000th generation. The posterior ESS was 148, the likelihood ESS 4171, the treeLikelihood COI ESS 12300, the treeLikelihood ITS ESS 2050. Trees and posterior probabilities were summarized using TreeAnnotator v. 2.7.3 and showed on the Maximum clade credibility tree, with a Posterior probability limit = 0.5 and Burnin percentage = 0.1. The trees were drawn in FigTree v.1.4.4 [44].

As another way to simultaneously analyze the two involved markers, ITS and COI, we made an alignment of both markers concatenated and analyzed it with partitioned substitution models for both genes via the Bayesian inference with MRBAYES and with the Maximum Likelihood Method with IQ-TREE. The MRBAYES tree reached convergence and had an ESS of 4665 for the combined runs, and the models for the partitions were for both the General Time Reversible (GTR) model with gamma-distributed rates across sites. For COI, the invertebrate mitochondrial code was used, and for ITS, the universal code was used. For IQ-TREE 2.3.5, we first estimated the best model of evolution for each partition, which was then run for 50,000 ultra-fast bootstraps. For COIm, the Tree-parameter Model (TPM2) model with empirical base frequencies, allowing a proportion of invariable sites, and the discrete Gamma model was selected; for ITS, the Hasegawa–Kishino–Yano (HKY) model was used with empirical base frequencies, allowing a proportion of invariable sites, and the Free Rate model was used with Rate heterogeneity across sites.

#### 2.2.2. Haplotype Analysis of *Anotogaster* spp.

POPART [45] was used to create the haplotype networks with the TCS (the method by Templeton, Crandall, and Sing, see [46]) network interference method from the COI alignment. A haplotype network shows the evolutionary sum of mutations that separate a given haplotype from other ones by connecting a current DNA molecule with the ancestral DNA molecule. 

We also performed a species delimitation test with the mPTP program using the multi-rate Poisson Tree Processes [47] for all *Anotogaster* specimens using *Aeshna grandis* as the outgroup. Four independent runs with each 1 billion mcmc generations were executed and sampled every 100,000 generations. The mPTP test shows congruence on the COI tree but not on the ITS tree.

## 3. Results

### 3.1. Phylogenetic Reconstructions

#### 3.1.1. Phylogenetic Reconstruction Based on the ITS Region

**Figure 1:**
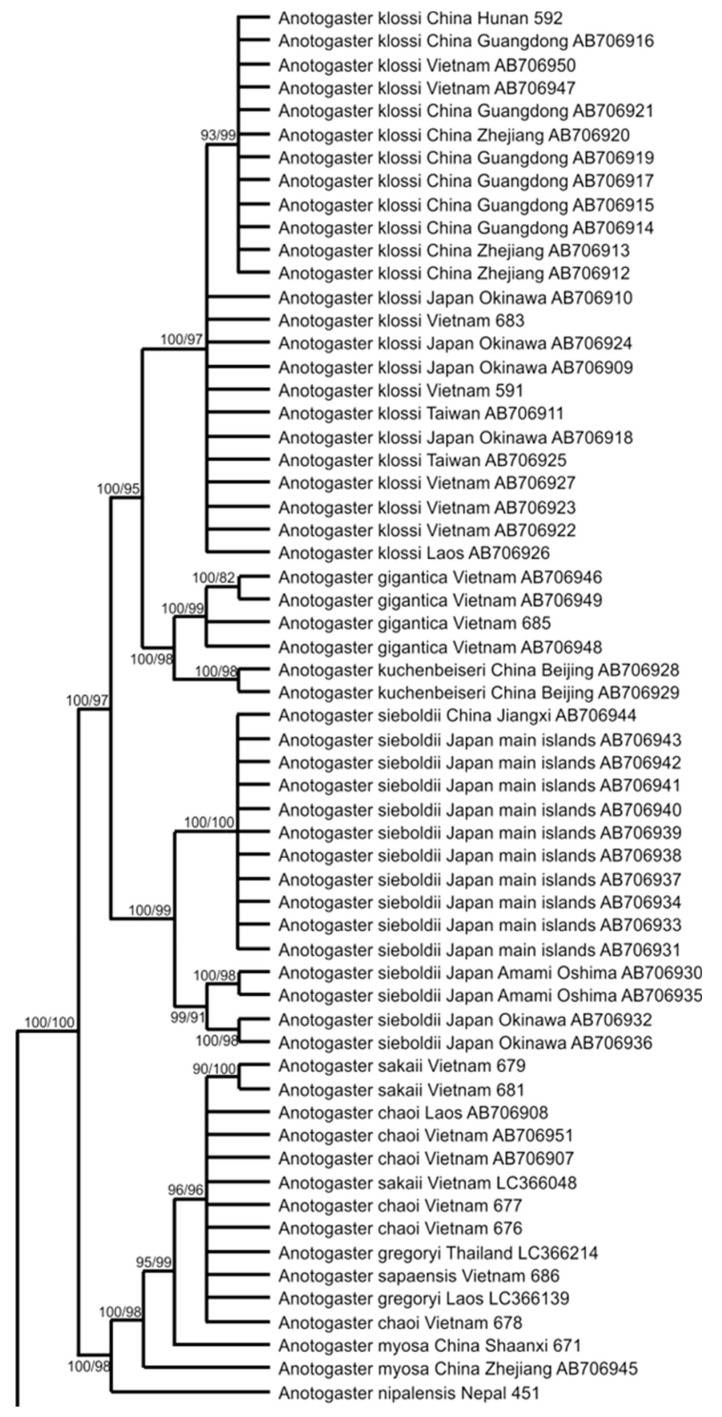

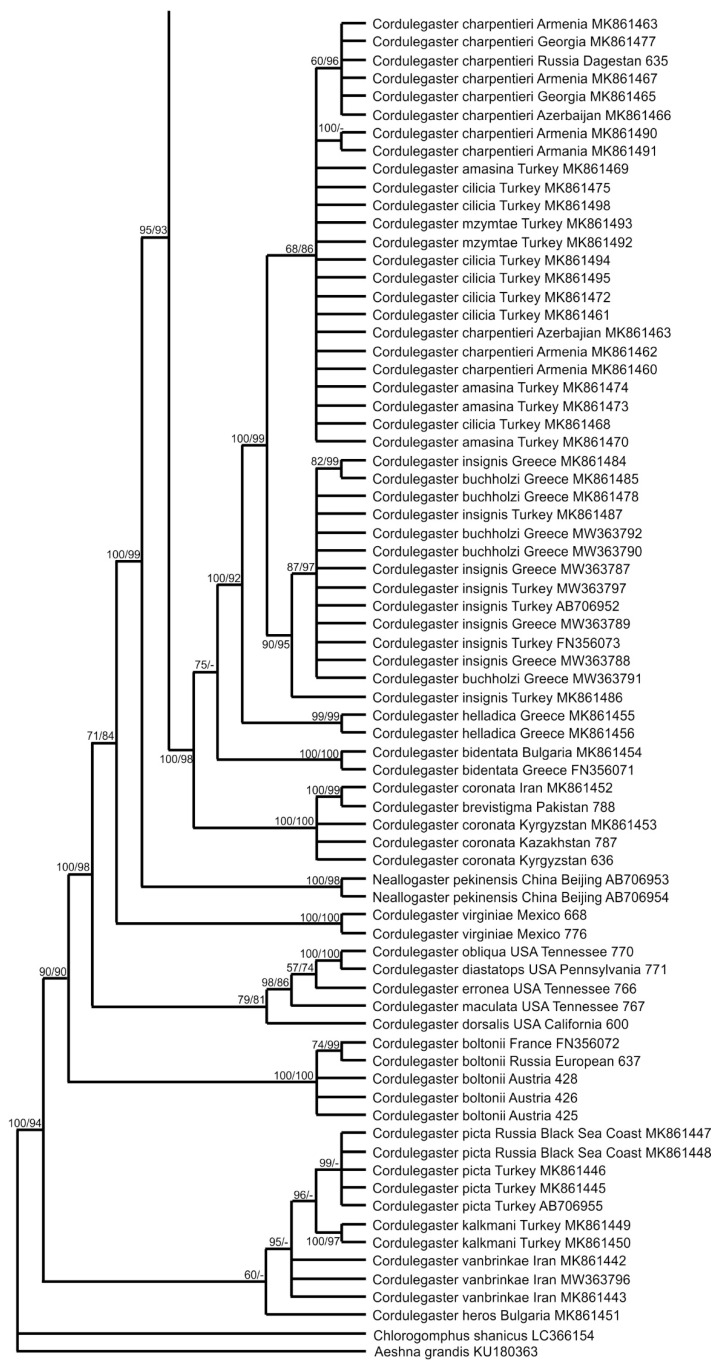
Phylogenetic tree inferred from sequences of the ITS region by Bayesian analysis with MRBAYES 3.2.7a using the best-fit model (HKY + G) identified with JMODELTEST 2.1.10. The values (×100) of the Bayesian posterior probability and (after a slash) ultrafast bootstrap values are provided at the nodes. Included are the sequences obtained in this study (DNA numbers next to the names) and those retrieved from GenBank (accession numbers next to the names).

We began our analysis with the sequence of the nuclear ITS region. The phylogenetic tree of Cordulegastridae reconstructed with the Bayesian method on its base is presented in Figure 1.

The family Cordulegastridae has maximum support against *Chlorogomphus shanicus*, a representative of another, although closely related, family Chlorogomphidae. The most basal nodes of the tree, except for the clade which contained all species of Cordulegastridae but *C. heros* Theischinger, 1979, and the cluster uniting *C. coronata*, i *C. brevistigma* Selys, 1854 and *C. bidentata* Selys, 1843, had high support above 0.85. It is convenient to describe the tree from the basal to crown branches, that is, from bottom to top. The most basal branch consisted of *C. heros*. The next clade included the rest of the species of the eastern *boltonii* group (*C. picta* Selys, 1854; *C. kalkmani* Schneider et al., 2021; *C. vanbrinkae* Lohmann, 1993). However, *C. boltonii* itself formed a separate clade. The next three clades consist of the American members of the family. The first of these three clades was represented by *C. dorsalis* Hagen in Selys, 1858; the next by *C. maculata*, *C. diastatops*, and *C. obliqua*; and the third by *C. virginiae.* The next clade consisted of *N. pekinensis*; it clustered with a high support of 0.96, with the large clade including the species of the *bidentata* group. The last clade corresponded to all members of the genus *Anotogaster*. 

The main biological message of the ITS tree was that the genus *Anotogaster* and the *bidentata* group of *Cordulegaster* (also including *N. pekinensis*) had been revealed as two very well-supported monophyletic clades. The American species in the ITS phylogenetic tree formed three subtle branches in the same basal cluster as the two last-mentioned large clades. Many species in the *bidentata* group were not resolved with the ITS region because of insufficient variation in the ITS region [5,14].

The *Anotogaster* clade deserves special consideration. It diverges into six sub-clades. The upper sub-clade (i) was represented by *A. klossi* Fraser, 1919, [48]; the next one (ii) by *A. gigantica* Fraser, 1924 [49], and *A. kuchenbeiseri* (Förster, 1899) [50]. The following three sub-clades (iii–v) included members so far recognized as *A. sieboldii* (Selys, 1854) from Japan and China. The major sub-clade (iii) included specimens from China and the main islands of Japan. The two smaller sub-clades (iv–v) referred to two specimens from Amami Oshima (the biggest island in the Amami Archipelago) and two specimens from Okinawa Island, respectively. The members of the first five sub-clades (i–v), *A. klossi*, *A. gigantica*, *A. kuchenbeiseri*, and *A. sieboldii*, also shared some common phenotypic characters: large yellow spots on the base of the mandible and the medioventral tooth (mvt) on the upper appendage seen in lateral view (with the exception of *A. klossi*). The lowermost sub-clade (vi) included the rest of the *Anotogaster* species, with *A. nipalensis* (Selys, 1854) slightly divergent from the rest species, *A. sakai* Zhou, 1988, *A. chaoi* Zhou, 1998, *A. sapaensis* Karube et al., 2012, and *A. gregoryi* Fraser, 1923, which were not well discriminated by the ITS analysis. In the members of sub-clade (vi), the mvt on the upper appendages was not seen from a lateral view, and the yellow spots on the sides of the mandible base were absent.

Another point, already recognized in the literature, is the separation of the western *C. boltonii* from the rest of the *boltonii* clade represented by the eastern species *C.picta*, *C. vanbrinkae*, and *C. kalkmani*. The separation of these two entities was already proposed by Verschuren on the basis of larval morphology [51] and later by molecular phylogenetic analyses [5,14]. However, *C. heros*, which is considered a member of the *boltonii* clade, curiously appeared in the ITS tree to be an outgroup for the rest of the family. 

The phylogenetic tree, reconstructed on the base of the ITS region using the Maximum Likelihood Method using IQ-TREE 2.3.5, appeared very similar to the Bayesian tree, so it is provided in Supplementary Figure S1.

#### 3.1.2. Phylogenetic Reconstruction Based on the COI Gene Fragment

**Figure 2:**
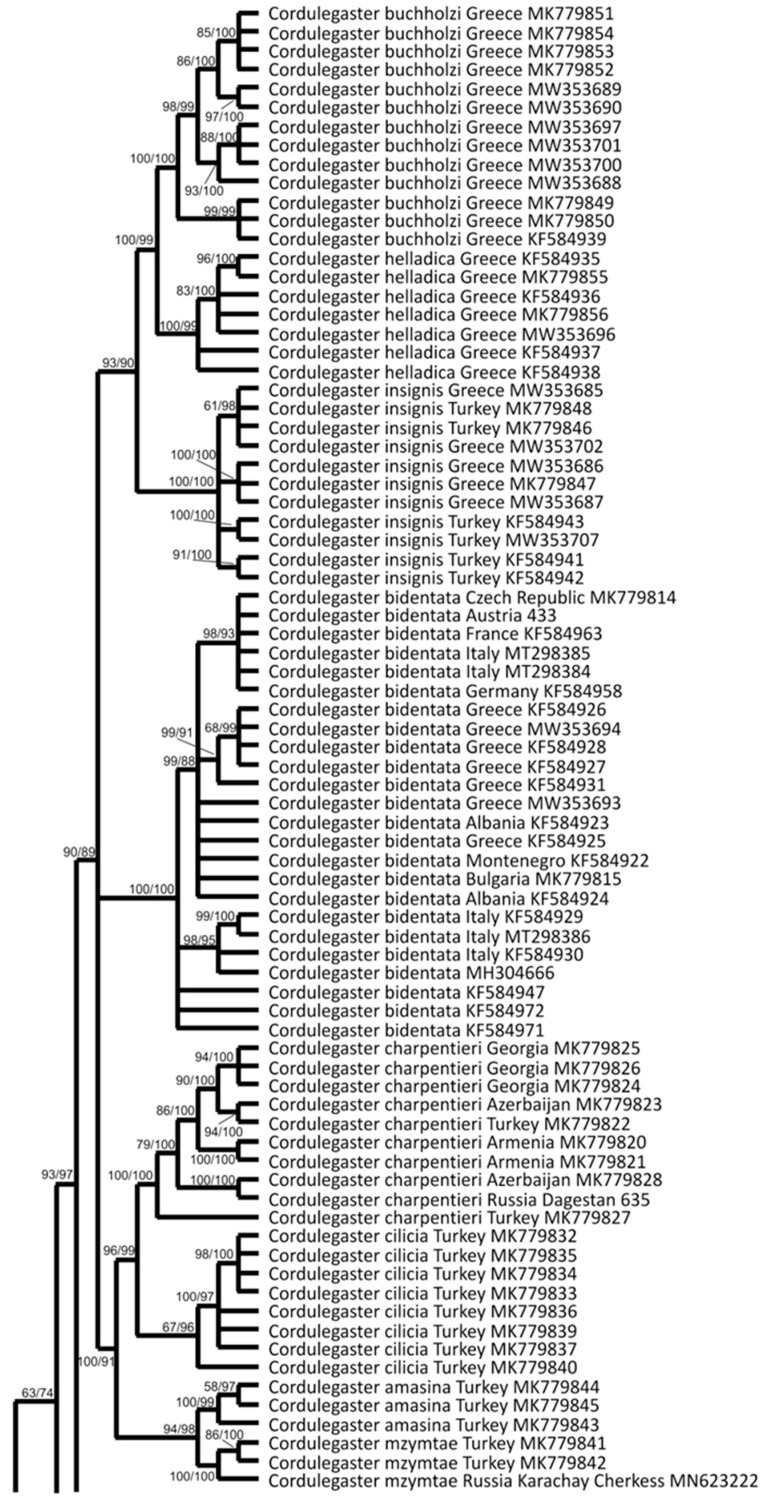

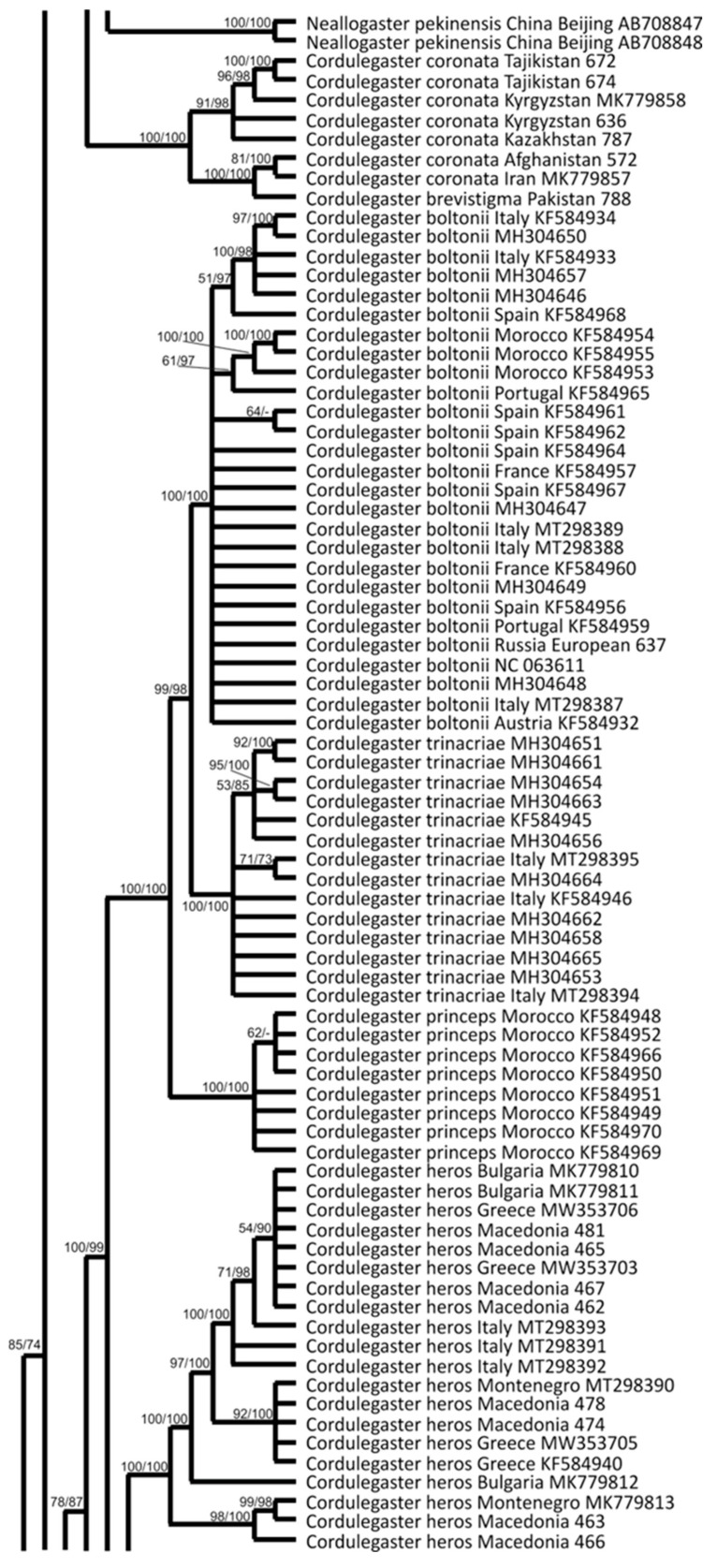

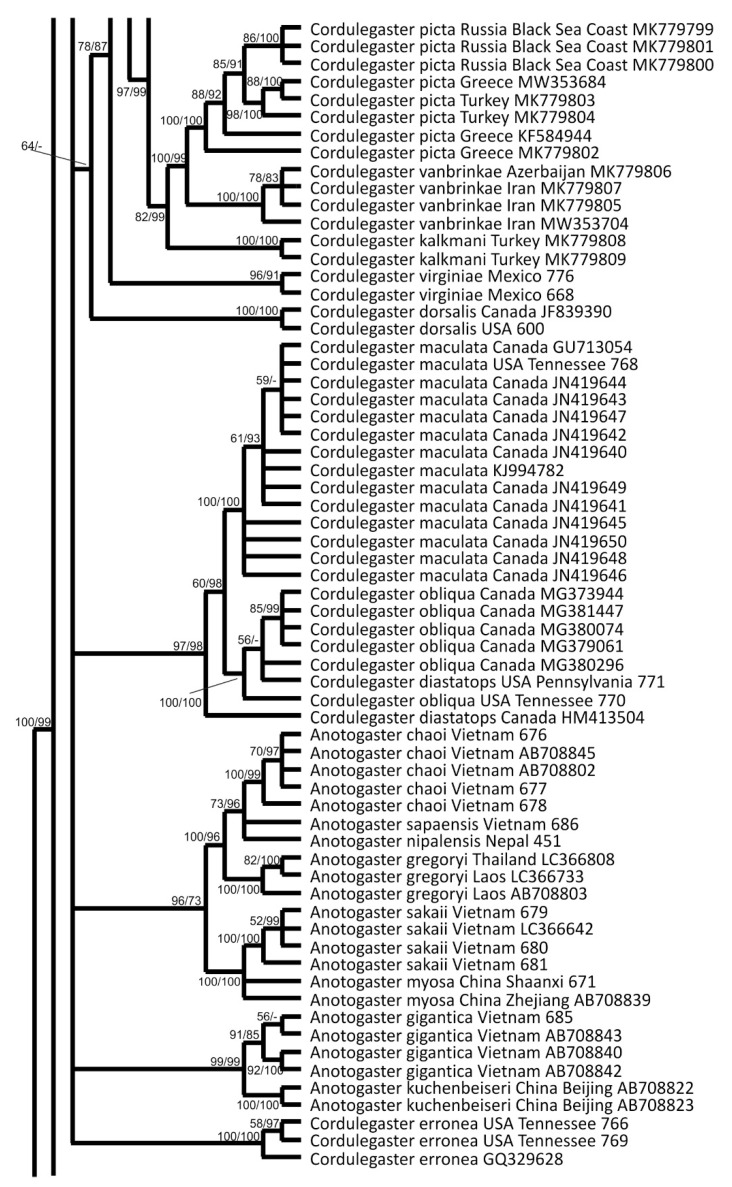

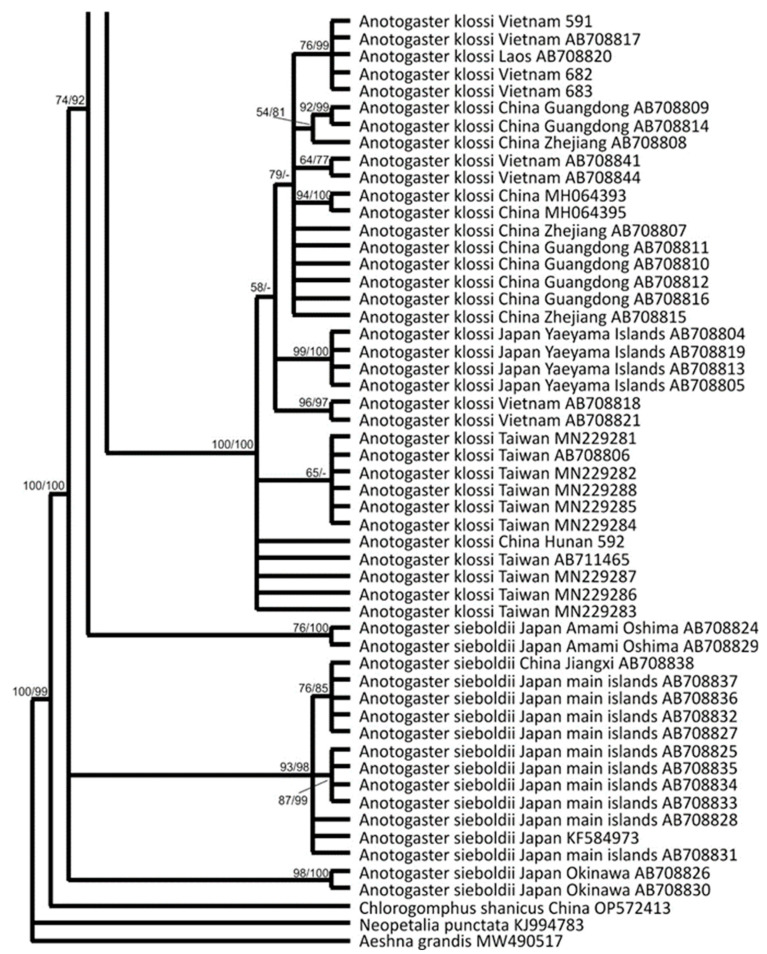
Phylogenetic tree inferred from the barcoding fragment of the mitochondrial COI gene by Bayesian analysis with MRBAYESs 3.2.7a using the best-fit model (GTR + I+G) identified with JMODELTEST 2.1.10. The values (×100) of the Bayesian posterior probability and (after a slash) ultrafast bootstrap values are provided at the nodes. Included are the sequences obtained in this study (DNA numbers next to the names) and those retrieved from GenBank (accession numbers next to the names).

The phylogenetic tree of Cordulegastridae reconstructed with the Bayesian method on the base of the barcoding COI gene fragment (further on the ‘COI tree’) is presented in Figure 2. It contains more sequences than the ITS tree. Concerning the included representatives of other families, it is recognized that the representative of Chlorogomphidae (*Chlorogomphus shanicus*) was nearer to the Cordulegastridae family than *Neopetalia* (Neopetaliidae), as expected.

As already discussed before [5,35,52,53], the COI analysis better resolves phylogeny at the species level than the ITS analysis but has some limitations in the higher taxonomic levels of genus and above. 

In the COI tree (Figure 2), *Anotogaster* spp. did not form a monophyletic clade but were split into six clades corresponding to the sub-clades of the monophyletic *Anotogaster* clade in the ITS tree (Figure 1). Remarkably, three of these clades were represented by specimens of *A. sieboldii* only (like in the ITS tree): one consisted of all members originating from Japanese main islands and eastern China, and two smaller clades contained the specimens from Okinawa and Amami, respectively. However, these three *sieboldii* clades in the COI tree were of the same rank as the sub-clade for the rest of the family. Thus, both analyses (ITS and COI) were in favor of three different allopatric species currently lumped under *A. sieboldii*. This view is also supported by the haplotype network analysis (see below). The formal description of the two taxa from Amami and Okinawa Archipelagos will be made elsewhere.

The next clade of *Anotogaster* consists of two closely related species, *A. kuchenbeiseri* and *A. gigantica*. Both share main phenotypic characteristics, such as large yellow spots on the mandible bases and the presence of a strong medioventral tooth (mvt) on the upper appendage. 

The next clade of *Anotogaster* consisted of all other species except for *A. klossi*. These six species (*A. nipalensis*, *A. sakai*, *A. chaoi*, *A. sapaensis*, *A. myosa* Needham, 1930, and *A. gregoryi*) are closely related, but most of them were well discriminated in the COI tree. Only *A. nipalensis* and *A. sapaensis* were not separated by the COI analysis. These two species are phenotypically very similar, having a reddish-brown face. Nevertheless, the haplotype network analysis (see below) supported the species level of these two. All six members of this clade have neither yellow spots on the mandible bases nor a conspicuous mvt on the upper appendages. The only described species missing from our analysis of *Anotogaster* is *A. basalis*, but based on its morphological characteristics, we would expect this species to also be in this clade.

The next clade of *Anotogaster* consisted of all specimens of the large geographic range of *A. klossi*. 

The next clade consisted of two sub-clades containing American species of *Cordulegaster*: one with *C. erronea* and the other with *C. diastatops*, *C. obliqua*, and *C. maculata*. Thus, the COI analysis was in favor of an extra clade for all these American species, which, however, does not include the southern species *C. virginiae* and *C. dorsalis*, which are closer to the old-world representatives of the *boltonii* group of *Cordulegaster*.

**Figure 3:**
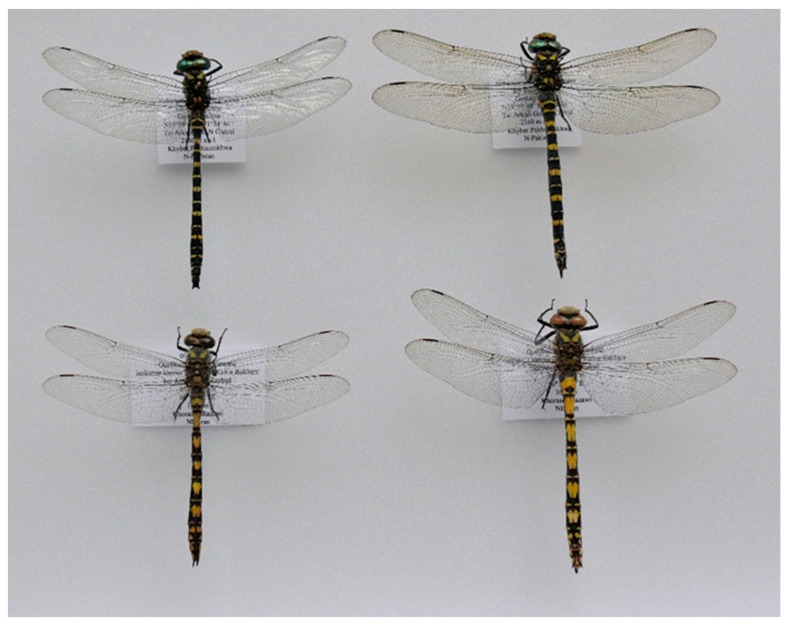
*Cordulegaster brevistigma*, above: ♂ left, ♀ right (Lutkho River, NW Chitral, Pakistan, 35.9966° N 71.5794° E, 2160 m a.s.l., 8.vii.2024, TS leg.). *Cordulegaster coronata*, below: ♂ left, ♀ right (Arzaneh, Iran, 34.9569° N 60.1672° E, 1683 m a.s.l., 11.vi.2016, TS leg.). Photo: Thomas Schneider.

The next large clade consisted of the *bidentata* group of *Cordulegaster*, *C. coronata*, and *Neallogaster pekinensis*. The discrimination at the species level with COI appeared much better than with ITS, as already shown before for the Western *Cordulegaster* species [5,14,35]. This clade was divided into five sub-clades, which were as follows, from the base to the top: (i) *C. coronata* and *C. brevistigma*, (ii) *N. pekinensis*, (iii) the *charpentieri* cluster including *C. charpentieri*, *C. cilicia* Schneider, et al., 2021, *C. amasina* Morton, 1916 and *C. mzymtae* Bartenev, 1929, (iv) *C. bidentata*, and (v) the *insignis*-cluster including *C. insignis*, *C. helladica* (Lohmann, 1993) and C. *buchholzi* (Lohmann, 1993). This topology was in line with previous studies [5,14,35]. Surprisingly, *C. coronata* and *C. brevistigma*, although looking so phenotypically different (Figure 3), appeared nearly genetically identical in both gene-fragment analyses (barcoding COI/Figure 2 and ITS/Figure 1), and further studies may reveal that *C. coronata* is just a subspecies or even a junior synonym of *C. brevistigma*.

The uppermost large clade included members of the *boltonii* group, as well as two American species, *C. virginiae* and *C. dorsalis.* It is remarkable that *C. virginiae* is, in fact, the southernmost *Cordulegaster* in America, occurring in Mesoamerica. The sub-clade of the *boltonii* group diverged into two well-supported branches, one representing the western members of the group (*C. boltonii*, *C. trinacriae* Waterston, 1976, and *C. princeps* Morton, 1916) and the other the eastern members (*C. heros*, *C. picta*, *C. vanbrinkae*, and *C. kalkmani*).

Again, the phylogenetic tree reconstructed on the basis of the COI using the Maximum Likelihood Method using IQ-TREE 2.3.5 appeared to have a similar topology to the Bayesian tree, as illustrated in Supplementary Figure S2 (a cladogram; the superficial differences are due to arbitrary rotation of nodes and do not concern topology).

#### 3.1.3. Haplotype Network Analysis of *Anotogaster* spp.

**Figure 4:**
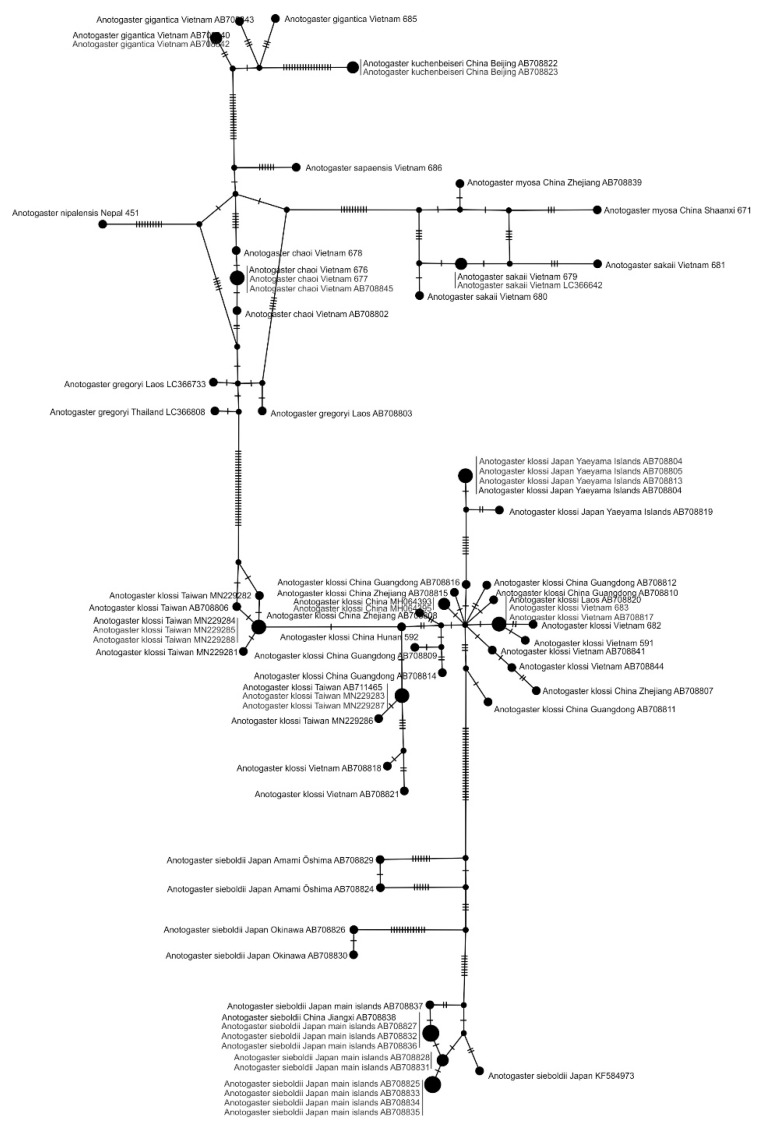
Haplotype network based on the barcoding fragment of the mitochondrial COI genes for *Anotogaster* spp., made in POPART 1.7 software. Hatch marks on the lines indicate the number of mutations.

To better understand the kinship relationships in the genus *Anotogaster*, we constructed a haplotype network for the studied COI fragments (Figure 4). In this network, the six sub-clades described above could be easily recognized as clusters: (1–3) *A sieboldii*; (4) *A. klossi;* (5) *A. nipalensis*, *A. myosa*, *A. sakai*, *A. chaoi*, *A. sapaensis*, and *A. gregoryi*; and (6) *A. kuchenbeiseri*, *A. gigantica*. The species within the genus were well separated, with the exception of *A. gregoryi* and *A. chaoi*. The latter two seemed to be closely related, which was also reflected in the phylogenetic trees of Figure 1 and Figure 2 (see above). *Anotogaster nipalensis* and *A. sapaensis* were better separated in the haplotype analysis than in the COI and ITS trees (Figure 1 and Figure 2). The separation of *A. sieboldii* specimens from the main Japanese islands and China from those originating from the Amami Oshima and Okinawa Islands was also well illustrated by the haplotype network (Figure 4). This analysis supported our identification of some unidentified sequences from GenBank [36] proposed on the basis of the phylogenetic trees we reconstructed (Table 2).

For all COI sequences of *Anotogaster* spp., we also performed a species delimitation test using the mPTP program with *A. grandis* as the outgroup. It revealed 11 species of *Anotogaster* (Supplementary Table S1). They corresponded to the recognized species, with the following exceptions: *A. myosa* and *A. sakaii* were recognized as the same species, while *A. sieboldii* from the main islands of Japan, Eastern China, Amami Oshima, and Okinawa were recognized as three different species, thus supporting the same inference from our other analyses.

#### 3.1.4. Species Tree (StarBeast Analysis)

To combine information from the two markers analyzed into a single species tree, we used the StarBeast 3 software specially designed for this purpose [41]. This approach takes into account that different DNA loci do not diverge alone but are incorporated into certain species, and these are species that actually diverge. To achieve this, this algorithm takes into account species identifications ascribed to sequences by biologists, taking them as the most probable assumptions of what the actual species are. Taking into account the divergence of *A. sieboldi* (see above), we subdivided the specimens of *A. sieboldi* into three conventional taxa, originating from the Okinawa, Amami, and main Japanese islands plus Eastern China, so that the program operated with them as with different species.

**Figure 5:**
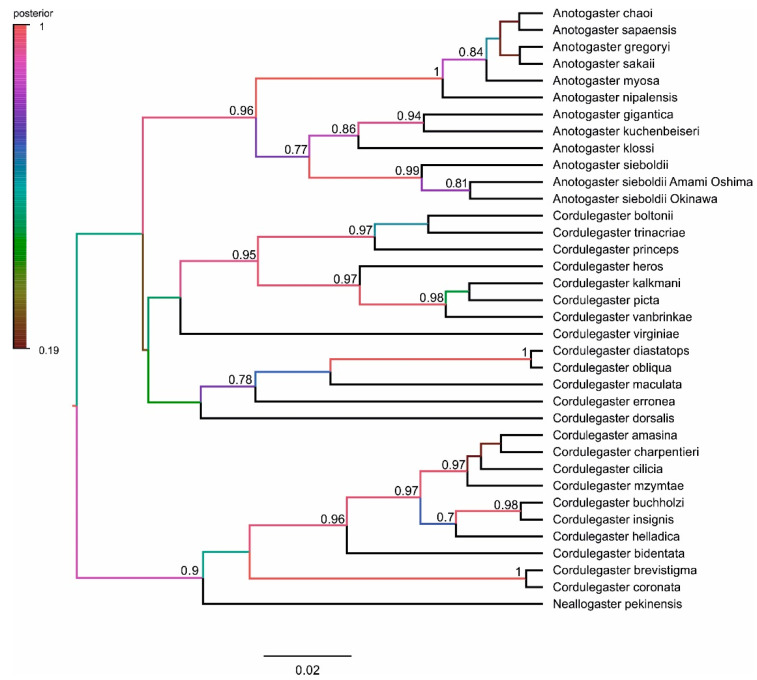
Multi-locus species tree reconstructed with StarBeast3 v 1.1.7 based on the COI gene fragment and the ITS region of representatives of Cordulegastridae. Bayesian posterior probability values (×100) are provided at the nodes (except for those below 0.7) and as colour in the branches.

The species tree reconstructed with StarBeast from the available COI and/or ITS sequences is shown in Figure 5. Since each species entered this tree only once, it was much easier to perceive directly. As expected, StarBeast analysis provided the best result at a supra-species level. 

First, StarBeast3 supports the current concept that the next relative to the family Cordulegastridae is the family Chlorogomphidae.

The StarBeast reconstruction summarised some important features of the ITS and COI trees. It suggests that the family Cordulegastridae can be divided into four major clades. 

Two of them, corresponding to the *boltonii* group and the *bidentata* group (again including *N. pekinensis*), had high support (0.85 and 0.98, respectively). The genus *Anotogaster* was also very well supported (0.9). These three major clades were monophyletic and corresponded to the current taxonomy. More weakly supported and more complex was the situation with the American members of the family. One clade with four American members, *C. diastatops*, *C. obliqua*, *C. maculata*, and *C. erronea*, could be assigned to the former genus *Zoraena*. *C. dorsalis* clustered with it as well but with negligible support. The Mesoamerican *C. virginiae* clustered with the *boltonii* clade of *Cordulegaster*, although with weak support.

The same StarBeast3 v 1.1.7 program also produced separate gene trees for each marker, provided in Supplementary Figures S3 (ITS) and S4 (COI). Again, the principal topology of these trees was similar to those of the respective Figure 1 and Figure 2, reconstructed by MRBAYES separately for these markers. However, some differences could be recognized. In the ITS gene tree (Figure S3), *C. boltonii* clustered together with other representatives of the *boltonii* group with maximum support, which better corresponded to the traditional systematic than their being two separate clades in Figure 1. Moreover, all six American species clustered together with a high support of 0.92 (not so in Figure 1). The topology of the StarBeast gene tree for COI (Figure S4) corresponded well to that resulting from the separate COI analysis by MRBAYES (Figure 2). In particular, the Amami and Okinawa specimens of *A. sieboldii* formed two basal clades, *C. dorsalis*, and *C. virginiae* formed independent subtle clades, and all American species of *Cordulegaster* clustered with the Old World *boltonii* group.

#### 3.1.5. Phylogenetic Analysis of Concatenated ITS and COI Sequences

**Figure 6:**
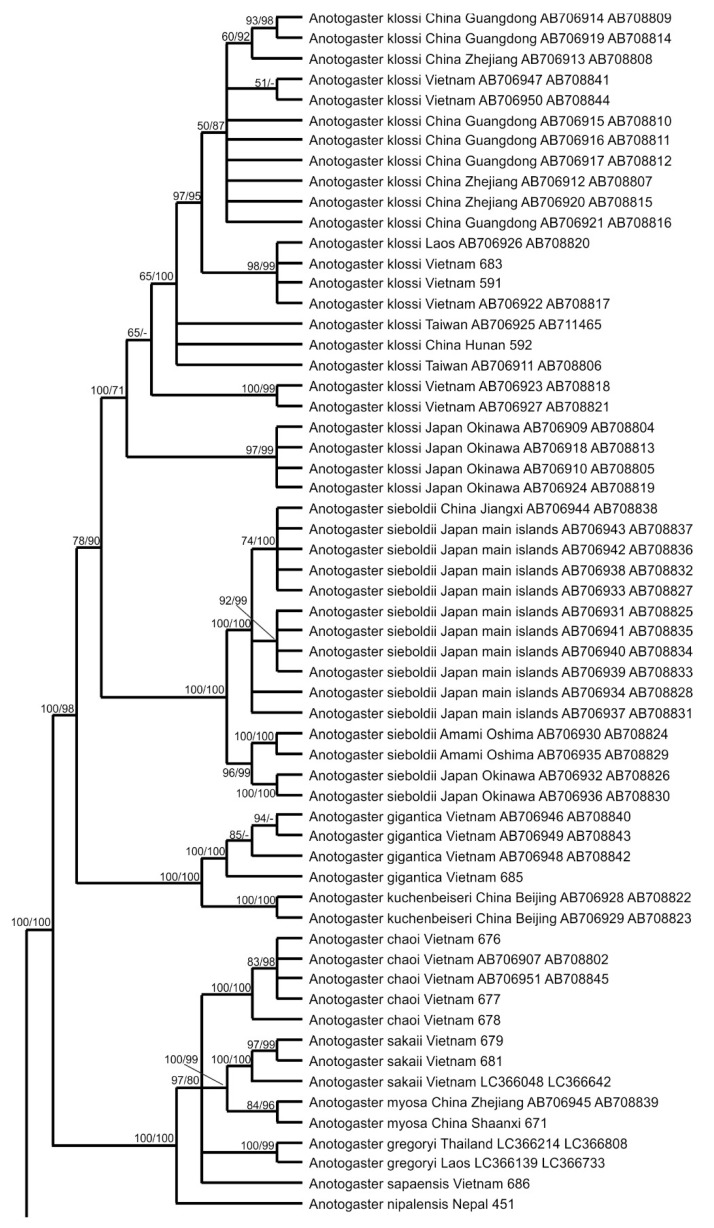

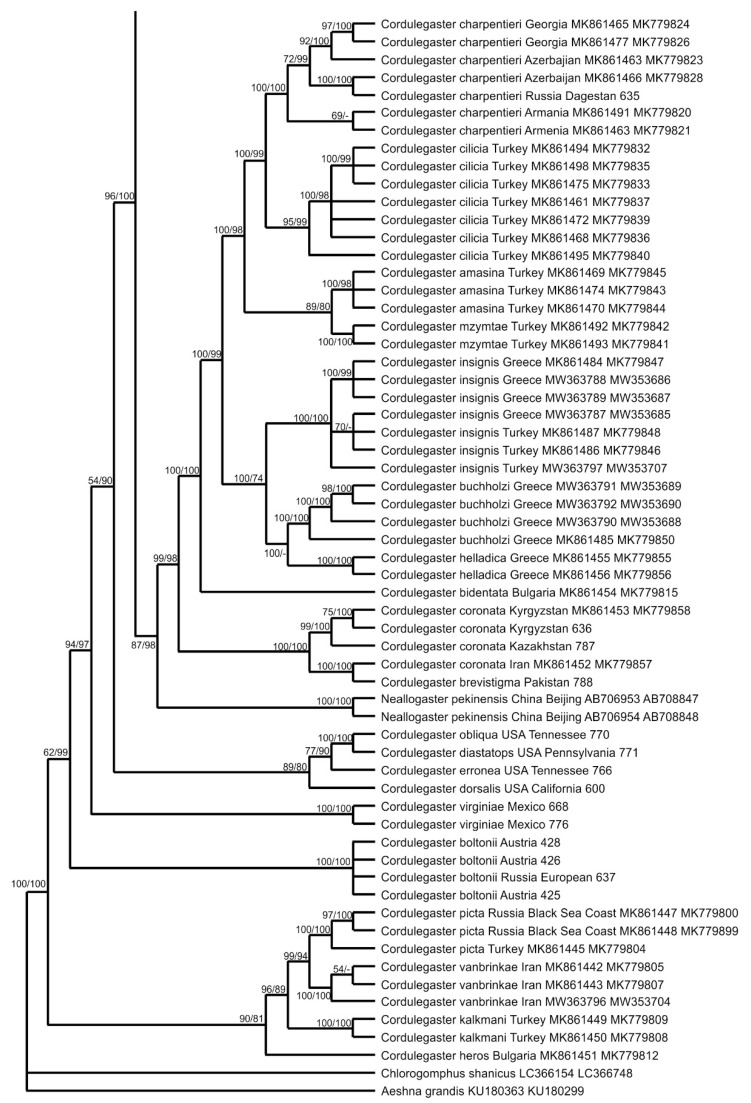
The phylogenetic tree reconstructed using MRBAYES 3.2.7 for the concatenated sequences of ITS region and COI gene fragment. The values (×100) of the Bayesian posterior probability and (after a slash) ultrafast bootstrap values are provided at the nodes. For the sequences obtained in this work, DNA numbers are provided. For those adopted from our previous works [5,35], the GenBank accession numbers for the ITS region and COI sequences are provided.

The analysis of concatenated ITS and COI sequences was limited to those specimens in which both markers were sequenced. These were mostly specimens analyzed by us in previous studies [5,35] and in this study. Since the concatenated analysis was another attempt, more straight-forward but less biologically justified (using an artificially constructed sequence), of a joint analysis of the two markers, it is logical to compare the resulted phylogenetic trees, reconstructed with the Bayesian (Figure 6) and Maximum Likelihood (Figure S5) methods with the species tree obtained by StarBeast (Figure 5). The phylogenetic trees based on the concatenated sequences reconstructed by both methods (Figure 6 and Figure S5) had the same topology, which resembled that of the species tree by StarBeast (Figure 5), but the following differences can be pointed out. *C. boltonii* formed a clade of its own that was not related to the rest of the *boltonii* group. *C. dorsalis* clustered with other American species in the Bayesian tree (Figure 6) or formed a separate lineage in the Maximum Likelihood tree (Supplementary Figure S5). Specimens of *A. sieboldii* did not cluster together but were found in two different subclusters of that species, one for the main Japanese islands and the other for Okinawa and the Amami Oshima Islands.

### 3.2. Doubtful Species of Anotogaster

Currently, 14 species are listed in the genus *Anotogaster* [1]. The majority of them were recorded from China and Vietnam. There is no general revision of this genus, although it was already emphasized by different odonatologists as badly needed [2,8,13,54]. As outlined above, the members of the whole family, especially of the genus *Anotogaster*, are very similar in the general habitus and the colouration patterns. The latter may vary in the same species, and even the male appendages may be similar in different species. Moreover, it is nearly impossible to separate species by comparing females. Therefore, it was absolutely mandatory to reconsider species in this family using molecular tools besides the traditional phenotypic characters [4,8,54]. H. Karube [54] and Karube et al. [4] have already resolved the puzzle of *Anotogaster* from Japan, Taiwan, and partially Vietnam. They clearly showed that the “*A. sieboldii*-clade” containing *A. sieboldii*, *A. kuchenbeiseri*, *A. klossi*, *A. antehumeralis*, and *A. flaveola* was confused due to incomplete original descriptions. They used the molecular phylogenetic approach and phenotypic characterization to resolve the status of Japanese and Taiwanese representatives of *Anotogaster*. They re-described in detail *A. klossi* and synonymized *A. flaveola* with *A. klossi* [4]. The rest of Lohmann’s species of the genus remained unresolved. 

Here, we would like to clarify those four doubtful species of *Anotogaster*, which were never reported again since their original description over 30 years ago and which were not properly compared with the already described species in the original descriptions. Since they are still represented by old-type specimens only, some of which were lost, we naturally could not investigate them by molecular means. Yet we find their true identity clear, as explained below.

**Figure 7:**
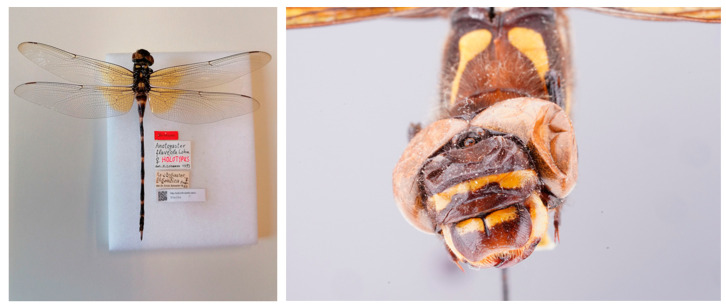
Holotype of *Anotogaster flaveola* (female) from Taiwan, V. Rolle leg., Museum für Naturkunde, Berlin [32]. Habitus (left) and front view (right). Photos: Birgit Jaenicke.

**Figure 8:**
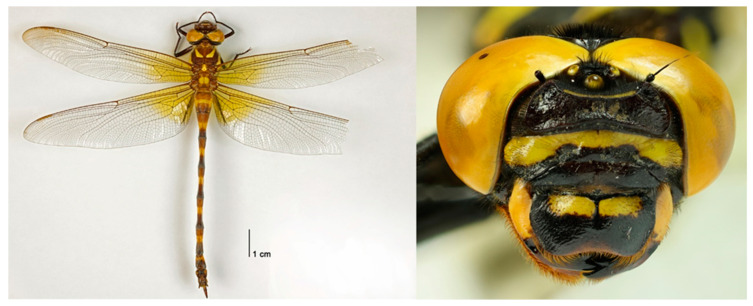
*Anotogaster klossi* females from Taiwan. Left: Taiwan: Yilan Co., Fushan, date unknown, leg. W. C. Yeh (No. 103278); Right: TAIWAN: New Taipei City, Gongliao, Hemei, 19.viii.1999, leg. W. C. Yeh (No. 103280). Both specimens are deposited at the Taiwan Forestry Research Institute, Taipei, Taiwan.

*Anotogaster flaveola* Lohmann, 1993, was described from a single female specimen (the holotype) from Taiwan (Figure 7). This specimen was found in the Natural Museum in Berlin (Museum für Naturkunde, Berlin). The name “*flaveola*” referred to the saffron colour patches at the wing bases (Figure 7). Such saffron colour patches at the wing bases are a common feature of juvenile females of all species of *Anotogaster* and will disappear in most individuals with maturation. Neither the original description nor the appearance of the specimen type distinguishes it from other females of *A. klossi* from Taiwan (Figure 8). The extensive morphologic and molecular genetic study by Karube et al. [4] clearly demonstrated that the only *Anotogaster* occurring in Taiwan is *A. klossi* and that *A. flaveola* is a junior synonym of *A. klossi.* This view is clearly supported by our investigation of the holotype.

*Anotogaster xanthoptera*, Lohmann 1993 was described from a single female specimen from Birma (Burma, Myanmar). This specimen type should be in the Museum König in Bonn, Germany. In response to our inquiry in December 2023, the curator of the Odonata collection, Dr. Dirk Gassmann, replied that the type cannot be found and is apparently lost. Neither the part of Myanmar where the type specimen was captured nor the date of collection was reported. The colour pattern of Odonata can change with age; for example, the wings may be more yellow-tinted in juvenile specimens, becoming more hyaline in older individuals. Thus, the name “*xanthoptera*” could indicate a teneral specimen. Moreover, after storage for a long time, the colours of a dead specimen may change depending on preservation conditions. Thus, the description of colours and patterns must be used with caution. As mentioned above, most juvenile *Anotogaster* females have saffron-tinted wings, which are more intense at the wing base. This is also known for *A. gregoryi* [8,55], which is widespread in South East Asia and reported in the neighboring countries, e.g., from North Thailand, close to the border of Myanmar [56]. The description of the head of the female given by Lohmann [32] also fits well with that of *A. gregoryi*: labrum with two large yellow spots, medially separated by a band, a black anteclypeus, a yellow postclypeus, and a brown mandible without yellow spots. These are good characteristics for differentiating *A. gregoryi* from *A. gigantica*, *A. kuchenbeiseri*, *A. klossi*, and *A. sieboldii*, which all have these yellow spots on the mandibles [8,48,49,50] and the black frons with a citron yellow stripe on the top (crest) (see also the original description by Fraser [7,55], repeated by him in Fraser [7]). Even in these two descriptions by Fraser, given six years one after another by the same author, some details varied; for example, the black colour turned brown, which may be due to storage in dissolved alcohol, as mentioned by Fraser [7]. The measurements that were given and the sketch of the ovipositor agreed well with those of *A. gregoryi*. Therefore, we suggest the following synonymy: 

*Anotogaster gregoryi* Fraser, 1923, valid name = *Anotogaster xanthoptera* Lohmann, 1993, syn. nov.

**Figure 9:**
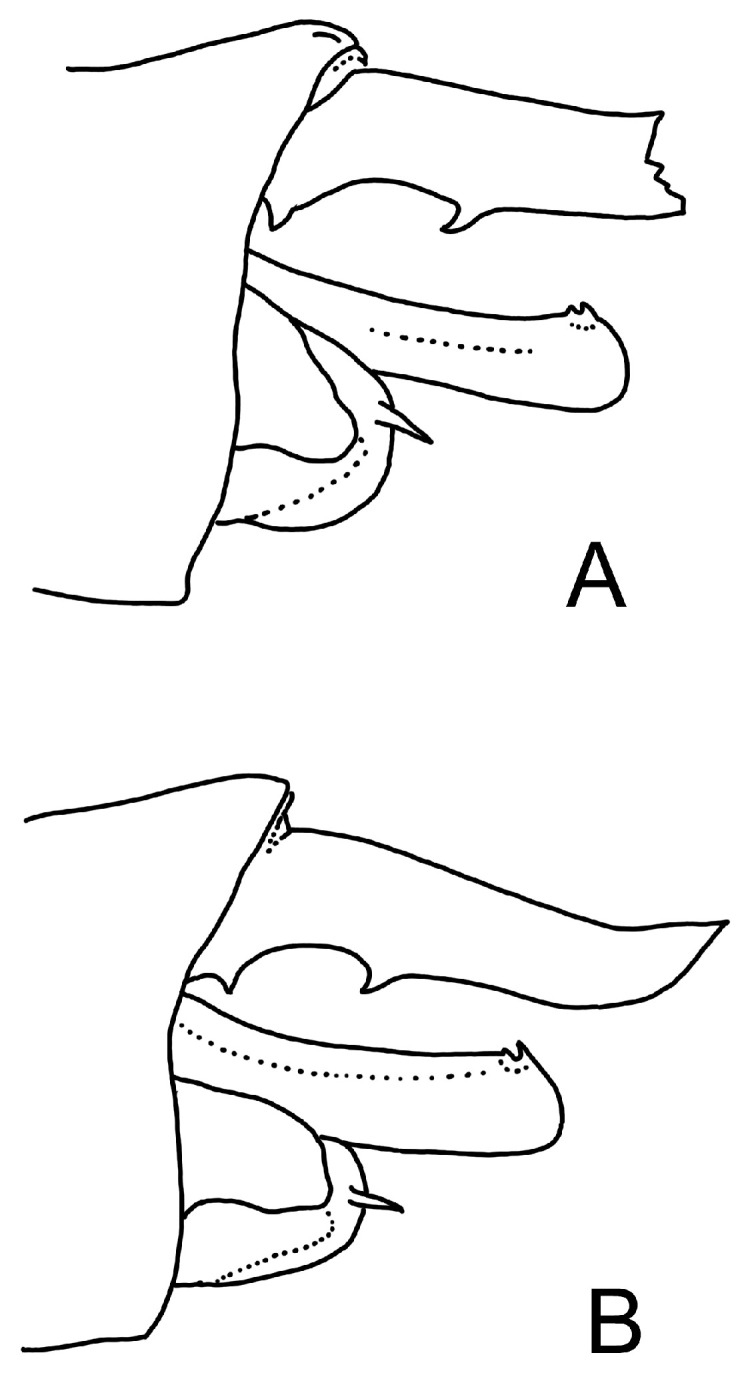
Sketches of the male appendages from lateral view: (**A**) redrawn from Lohmann (Figure 14 inside [32]) *Anotogaster cornutifrons*; (**B**) redrawn from Zhang [8], page 580 *Anotogaster kuchenbeiseri*. Artwork: Ole Müller.

*Anotogaster cornutifrons*, Lohmann 1993 was described from the male holotype and male and female paratypes, all collected in June 1936 in Central China, Shaanxi, and deposited in the Museum in Leiden (now Naturalis Biodiversity Center). Neither the holotype nor the paratypes could be found in Naturalis (pers. comm. by Charlotte Hartong, the curator of Odonata in Leiden, Naturalis). However, she managed to find some specimens of *Anotogaster* from China, which we compared with the known *Anotogaster* spp. The pictures revealed another species, as the upper appendage had no medioventral tooth (mvt), as shown in the figure in Lohmann’s description. Thus, we had to refer to the original description. Fortunately, there was a further description of a female of this species by van Pelt [2], in which the frontal view of the head of a female was depicted ([2], Figure 1). Van Pelt already mentioned the variability of the colour pattern and the similarity with *A. kuchenbeiseri* [2]. Taking the measurements together, the male upper appendages with mvt (Figure 9, see also an original description of *A. kuchenbeiseri* by Förster [50]), the frontal pattern of the head of the female [2], and the location, there is no doubt that *A. cornutifrons* is a junior synonym of *A. kuchenbeiseri*:

*Anotogaster kuchenbeiseri* (Förster, 1899), valid name = *Anotogaster cornutifrons* Lohmann, 1993, syn. nov.

It should be stressed that our analysis does not support the synonymy of *A. kuchenbeiseri* to *A. sieboldii*, as suggested in the current World Odonata List [1]. 

**Figure 10:**
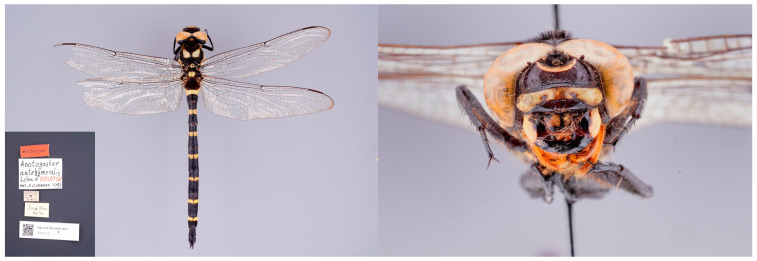
Holotype male of *Anotogaster anthehumeralis*, from Xinjiang Province, West China, the northern slope of Kunlun Mountains, “Tschakar bei Pulu (Polu), 1930 NN, 36°10′N 81°29′E”, 1930 m, 36°10′ N, 81°29′ E, 8.-10.vi.1890, S. Conradt leg., Museum für Naturkunde, Berlin [32]. Habitus dorsal view with the original label (**left**) and head frontal view (**right**). Photos: Birgit Jaenicke.

*Anotagaster antehumeralis* Lohmann, 1993 was described from a single male holotype allegedly collected in Western China, Xinjiang Province, at the northern foot of the Kunlun Mountains, “Tschakar bei Pulu (Polu), 1930 NN, 36°10′ N 81°29′ E”, in 1890 by S. Conradt. However, the powerful river flowing through the gorge in the Taklamakan Desert zone does not fit well with the known habitats of *Anotogaster*, so the old label may have been confused, and the specimen possibly originated from elsewhere in China. The holotype (Figure 10) was located by us in the Natural History Museum in Berlin, as stated in the original description. The specimen is smaller than *A. sieboldii* and is within the size range of *A. kuchenbeiseri* (abdomen plus cerci 62.8 mm, hindwing 44.4 mm). Its overall appearance and colour pattern with broad antehumeral stripes correspond again to *A. kuchenbeiseri*. The upper appendages have strong laterobasal teeth and medioventral tubercles (mvt); the latter is not easily seen from the side but is better from above (note that the cerci are mobile and can be rotated in different positions). The frontal pattern of the head shows all the characteristics of *A. kuchenbeiseri*: the top of the frons with a broad yellow concave stripe; the majority of the labrum is missing, as mentioned by Lohmann, but with remnants still showing rests of the yellow spots; the base of the mandible with large yellow spots; the yellow postclypeus (Figure 10). *Anotogaster kuchenbeiseri* is widely distributed in China and is known from Beijing, Shanxi, Shaanxi, Henan, Hubei, and Sichuan [8]. Thus, the specimen fits well with *A. kuchenbeiseri*, now better known with more details of the phenotype than at the time of Lohmann’s description of *A. antehumeralis*. Thus, taking all characters together, especially the characteristic frontal pattern of the face, the following synonymy is proposed:

*Anotogaster kuchenbeiseri* (Förster, 1899), 1924, valid name = *Anotogaster antehumeralis* Lohmann, 1993, syn. nov.

## 4. Discussion

### 4.1. Coverage of the Study

We were able to involve sequences from 34 of the 52 described species of the family Cordulegastridae, including all accepted genera so far [1]. If we remove the above-identified synonyms in the genus *Anotogaster*, the number is reduced to 48. Furthermore, *Cordulegaster parvistigma* Selys, 1873 was deleted from the list because of several reasons: the main description was based on a female (now lost), and Selys himself placed it near *C. brevistigma* ([57], pp. 64–65). This species has not been seen again since the original description until now; thus, it has been over 150 years. Therefore, we suggest synonymizing *Cordulegaster parvistigma* Selys, 1873 syn. nov. with *T. brevistigma* Selys, 1854 comb. restaur; so, the total number of Cordulegastridae is currently 47. Thus, we have analyzed over 70% of all known species of the family and all of the genus *Cordulegaster* in the current sense of the old world. 

For the current genus *Neallogaster*, a future thorough revision based on molecular data and morphological characteristics is needed to reveal possible synonyms and the exact phylogenetic positions of the remaining members currently placed in this genus. The transfer of *Cordulegaster pekinensis* to the genus *Neallogaster* by Lohmann [12] was already questioned by van Pelt [13]. Several morphological differences can be noted between *N. pekinensis* and other species currently considered in this genus. Its overall appearance of a bigger and more robust Cordulegastridae is in contrast to the other members of the genus. There are further striking phenotypical differences: it is black, without a brown tint on the underside of the abdomen, the abdominal yellow spots are larger than in other members of the genus, the thorax is not very hairy, the face is black and yellow without brown, the frons is not much inflated, the head is not very broad, the femora are not brownish, the legs are as long as other Cordulegastridae, the pterostigma is not shorter than in other Cordulegastridae, the anal triangles have three–four rather than two cells, and females have no dark anterior stripe along the costal wing margins. All these morphological characteristics of *pekinensis* fit more to members of the *bidentata* group. Moreover, in contrast to the other members of *Neallogaster*, they do not occur at such high altitudes (>2000 m a.s.l.). Thus, the placement of *pekinensis* in the genus *Thecagaster* based on molecular analysis is also supported by phenotypical and ecological characteristics.

### 4.2. Phylogeny and Generic Structure of the Family

As expected from our previous studies on the genus *Cordulegaster* [5,35] and the family Aeshnidae [52], as well as from many works by others on Odonata [58,59,60,61] and other orders, e.g., Orthoptera [53], the COI gene fragment analysis showed a better differentiation on the species level (barcoding) and therefore contained more sub-branches than the ITS analysis. The four major clades, in general, fit already known genera but also provided some new surprising insights, such as the clustering of *Neallogaster pekinensis* in the *bidentata* group. Surprisingly, *C. brevistigma* revealed a sister species of *C. coronata*. Moreover, further studies may reveal that *Thecagaster coronata* comb. nov. is indeed a subspecies or even a junior synonym of *T. brevistigma* comb. nov.

On the species level, all species analyzed with the molecular phylogenetic approach were confirmed, at least in the COI tree or haplotype analysis. However, *Anotogaster sieboldii* appeared more heterogeneous, as represented by three clades, indicating long isolation on Japanese islands. Thus, three taxa can be recognized, *A. sieboldii* and two unnamed species, one from Okinawa and one from Amami Oshima, as already suggested before [4].

The unidentified sequences from GenBank [36] could be identified at the species level (Table 2). The unidentified *Anotogaster* AB708839 (COI) and AB706945 (ITS) from China (Zhejiang) clustered in the COI tree (Figure 2) and haplotype analysis (Figure 4) together with a further unidentified *Anotogaster* 671 (COI) from China (Shaanxi). We were able to acquire one such specimen (from Qin Ling Mountains, Shaanxi, the number 671, which revealed the COI sequence). Its identification by comparing with the descriptions by Needham [62] and Zhang [8] revealed *A. myosa*. Thus, finally, we were able to assign all unidentified specimens from the GenBank [36] to a particular species.

We were able to synonymize the three *Anotogaster* species described by Lohmann [32] but never reported again since the original descriptions. Only *A. flaveola* was once reported, with a question mark by Asahina [31] from Vietnam and by Wilson [63] from Guangdong, China. However, subsequent studies, including molecular phylogenetic ones, clearly have shown that the Taiwanese population and the nearby southern Japanese island population of Yaeyama, as well as those from China, South Vietnam (type), and Laos, belong to the same species *A. klossi* [54,64,65]. *Anotogaster xanthoptera* was described based on a single female [32]. Despite an intensive search by the curator of the Odonata collection at the Museum König in Bonn (Dr. Dirk Gassmann), this type of female could not be found and was considered lost. We had to compare the description with the available literature, which revealed that the description was well in agreement with those of *A. gregoryi*. Thus, *A. xanthoptera* is indeed a junior synonym of *A. gregoryi*. The other two *Anotogaster* described by Lohmann [32] are from China. In the case of *A. antehumeralis*, the male type was located in the Museum of Natural History in Berlin. This specimen has a medioventral tooth (mvt) on the upper appendages, as in *A. kuchenbeiseri*, and the pattern of the frontal head with yellow spots on the mandible, as well as the measurements, also match *A. kuchenbeiseri*. Thus, we suggest *A. antehumeralis* to be a junior synonym of *A. kuchenbeiseri*. In the case of *A. cornutifrons*, the types and paratypes are lost (as communicated by Charlotte Hartong, Naturalis, Leiden). The supposed specimens of this taxon from the same Chinese province revealed, after careful examination, that they belong to another species (for example, no mvt as in Lohmann’s description). Thus, we had to refer to Lohmann’s original description and the re-description by van Pelt [2]. The structure of the male appendage and the pattern of the frontal head (very nicely depicted by van Pelt [2]) revealed, without any doubt, that *A. cornutifrons* is a synonym of *A. kuchenbeiseri*.

The two phylogenetic markers analyzed, ITS and COI, and, most importantly, the combined StarBeast3 (Figure 5) species tree and the tree for the concatenated sequence (Figure 6), support the division of the family Cordulegastridae in four clades, which can be attributed to four genera. The species attributed to *Anotogaster* form a monophyletic group in the ITS and joint sequence analyses, so this genus was confirmed by our study. Some of the species in this genus may further appear to be synonyms, for example, *A. nipalenis* and *A. sapaenis*. In the case of *A. sieboldii* and *A. klossi*, many problems were already resolved by the extensive study by Karube et al. [4]. We could locate the types of two of Lohmann’s species of *Anotogaster*; in the other two cases, we had to refer to the original description. After comparing the holotype with the available literature, we could confirm the conclusions of Karube et al. [4] that *A. flaveola* is a synonym of *A. klossi*. Furthermore, we synonymize *A. cornutifrons* and *A. antehumeralis* with *A. kuchenbeiseri* and *A. xanthoptera* with *A. gigantica*, thus reducing the species in this genus from 14 to 10. Our molecular analysis involved nine of these remaining ten species; only *A. basalis* was missing.

The StarBeast3 species tree (Figure 5), as well as the tree based on the concatenated sequences (Figure 6), resolves an acceptable version of the phylogeny of the Cordulegastridae family. As well as the ITS tree (Figure 1), it suggests the species attributed to the genus *Anotogaster* to form a monophyletic group. It also presents both Palaearctic groups of *Cordulegaster* as natural monophyletic groups, the *bidentata* group and *boltonii* group. However, the six American species were not found together in one clade. While *C. virginiae* loosely clustered with the *boltonii* clade, the rest formed a separate clade, which we associated with the already existing generic name *Zoraena* [30]. The generic attribution of *C. virginiae* and, very tentatively, *C. diadema* (which we did not analyze but which is related to *C. virginia* [10]) is problematic. For the time being, we tentatively left them in the genus *Cordulegaster*, in the narrow sense of this paper, together with the *boltonii* clade. However, some of the tree versions reconstructed by different methods and/or from different sequences (see above) do not support this attribution. It is not excluded that future molecular analyses based on more markers or a better genomic approach would suggest a separate genus for these three enigmatic American species.

According to our results, *Anotogaster* appears to be a valid genus in the current sense. The type species of *Cordulegaster* is *C. boltonii*, so the name *Cordulegaster* automatically denotes the monophyletic genus, which includes the *boltonii* group. The generic name *Zoraena* is available for the American species, except for *C. virginiae* (and perhaps *C. diadema*). 

For the genus referring to the clade formed by the *bidentata* group of *Cordulegaster*, the genus *Thecagaster* ([12] pp. 9–12), [21], ([66] pp. 587–589) is restored, which includes all members of the old-world *bidentata* group, including *T. coronata* comb. nov. and *T. brevistigma* comb. restaur., and also *T. pekinensis* comb. nov.. The genus *Neallogaster* needs further studies involving molecular and phenotypical approaches to see if it really exists or if its members will be distributed to other genera. The genus *Thecagaster* is characterized by the two teeth on the upper appendages, basal and medioventral ones, as, for example, in the well-known species *T. bidentata* or T. *insignis*. This feature is also strikingly present in *T. pekinensis* comb. nov.. Moreover, all the members of this genus share a unique preference for small trickles, spring waters, and seepages in mountain areas.

### 4.3. Preliminarily Proposed System of Cordulegastridae

The system of the family which follows from our data is provided below (see also Table 3 for taxonomic solutions). The boldfaced species were analyzed by us; others were tentatively attributed to genera only for the time being based on their morphological proximity to the studied species, e.g., as stated for American species by Abbott and Hibbitts [20].

Genus *Anotogaster* Selys, 1854

*A. basalis* Selys, 1854; ***A. chaoi*** Zhou, 1998; ***A. kuchenbeiseri*** (Förster, 1899) (=*cornutifrons*, Lohmann 1993 syn. nov.; =*antehumeralis*, Lohmann 1993 syn. nov.); ***A. gigantica*** Fraser, 1924; ***A. gregoryi*** Fraser, 1923 (=*xanthoptera* Lohmann 1993 syn. nov.); ***A. klossi*** Fraser, 1919 (=*A. flaveola* Lohmann 1993 syn. confirm.); ***A. myosa*** Needham, 1930; ***A. nipalensis*** (Selys, 1854)*; **A. sakaii*** Zhou, 1998; ***A. sapaensis*** Karube, 2012; ***A. sieboldii*** (Selys, 1854).

Genus *Cordulegaster* Leach in Brewster, 1815

***C. boltonii*** (=*annulata* Latreille, 1805; =*forcipata* Harris, 1780; =*grandis* Scopoli, 1763; =*lorenzoni* Disconzi, 1865; =*lunulata* Charpentier, 1825; =*orientalis* van Pelt, 1994); *C. diadema* (Selys, 1863) (=*godmani* McLachlan 1886, tentatively); ***C. heros*** Theischhinger, 1979; ***C. kalkmani*** Schneider, Vierstraete, Muller, van Pelt, Caspers, Ikemeyer, Snegovaya & Dumont, 2021; ***C. picta*** Selys, 1854; ***C. princeps*** Morton, 1916; ***C*. *trinacriae*** Waterston, 1976; ***C. vanbrinkae*** Lohmann, 1993; ***C. virginiae*** Novelo-Gutiérrez, 2018.

Genus *Thecagaster* (=*Allogaster* Selys, nom. praeocc.; =*Sonjagaster* Lohmann, 1992; =*Kuldanagaster* Yousuf & Yunus, 1974) 

***T. amasina*** (Morton, 1916) comb. nov., ***T. bidentata*** (Selys, 1843) comb. nov., ***T. brevistigma*** Selys, 1954 comb. restaur. (=*T. parvistigma* Selys, 1873 = *pakistanica* Yousuf & Yunus, 1974); ***T. buchholzi*** (Lohmann, 1993) comb. nov., ***T. charpentieri*** (Kolenati, 1846) comb. nov. (=*nachitschevanica* Skvortsov & Snegovaya, 1915; =*plagionyx* Skvortsov & Snegovaya, 1915); ***T. cilicia*** (Schneider, Vierstraete, Müller, van Pelt, Caspers, Ikemeyer, Snegovaya & Dumont, 2021) comb. nov., ***T. coronata*** (Morton, 1916) comb. nov., ***T. helladica*** (Lohmann, 1993) comb. nov., ***T. insignis*** (Schneider, 1845) (=*magnifica* Bartenev, 1930; =*montandoni* St. Quentin, 1971) comb. nov.; ***T. mzymtae*** (Bartenev, 1929) comb. nov.; ***T. pekinensis*** (McLachlan in Selys, 1886) comb. nov.

Genus *Zoraena* Kirby, 1890 (=*Thecagaster* Selys, 1854, nom. praeocc.)

***Z. bilineata*** Carle, 1983 comb. restaur.; ***Z. diastatops*** (Selys, 1864) comb. restaur. (=*lateralis* Scudder, 1866); ***Z. dorsalis*** (Hagen in Selys, 1853); ***Z. erronea*** Selys, 1878 comb. nov.; *Z. sarracenia* (Abbott & Hibbitz, 2011) comb. nov.; ***Z. maculata*** (Selys, 1854) comb. nov.; ***Z. obliqua*** (Selys, 1844) comb. nov. (=*fasciatus* Rambur, 1842); *Z. sayi* (Selys, 1854) comb. nov.; *Z. talaria* Tenessen, 2004 comb. nov. 

The above system is based on molecular evidence, and the morphological diagnoses of four genera are well established. However, *Neallogaster*, in the old sense, remains to be unraveled. We placed *T. pekinensis* comb. nov. in the genus *Thecagaster*. It has some morphological differences from the true members of the genus *Neallogaster*, as outlined above. The content of *Anotogaster* did not change, as well as its diagnosis being the absence of the auricles and anal triangles in males. *Thecagaster* differs from *Cordulegaster* by two teeth, rather than one tooth, on the cercus. The members of this genus also share a common preference for small trickles, spring waters, and seepages in mountain areas. The phenotypic differences of *Zoraena* from *Cordulegaster*, in their new senses, are not well established and more or less the same as between the “*diastatops*” clade and the rest of American *Cordulegaster* according to Abbott and Hibbitts [20]. According to these authors, the species of *diastatops* clade, that is, *Zoraena* in our sense, share a unique combination of characteristics, including male epiproct beyond S10 wider than long, male cerci separated basally by more than twice the basal width of cerci, male cerci inflated beyond the ventral spine, compound eyes distinctly separated dorsally, posterior surface of eyes with a tumid, and vulvar lamina extending approximately half its length beyond the cerci [20].

## Figures and Tables

**Table 3:** A summary of synonymizations at species rank and new combinations.

Currently Used Names	Valid Name According to This Paper
***synonymization at species rank***	
*Anotogaster xanthoptera* Lohmann, 1993 **syn. nov.**	*Anotogaster gregoryi* Fraser, 1923
*Anotogaster flaveola* Lohmann, 1993 **syn. confirm**.	*Anotogaster klossi* Fraser, 1919 (see [4])
*Anotogaster antehumeralis* Lohmann 1993 **syn. nov.**	*Anotogaster kuchenbeiseri* (Förster, 1899)
*Anotogaster cornutifrons* Lohmann, 1993 **syn. nov.**	*Anotogaster kuchenbeiseri* (Förster, 1899)
**new combinations**	
*Cordulegaster amasina* Morton, 1916	*Thecagaster amasina* (Morton, 1916) **comb. nov.**
*Cordulegaster bidentata* Selys, 1843	*Thecagaster bidentata* (Selys, 1843) **comb. nov.**
*Cordulegaster brevistigma* Selys, 1954	*Thecagaster brevistigma* (Selys, 1954) **comb. restaur.**
*Cordulegaster buchholzi* (Lohmann, 1993)	*Thecagaster buchholzi* (Lohmann, 1993) **comb. nov.**
*Cordulegaster charpentieri* (Kolenati, 1846)	*Thecagaster charpentieri* (Kolenati, 1846) **comb. nov.**
*Cordulegaster cilicia* Schneider et al. 2021	*Thecagaster cilicia* (Schneider et al. 2021) **comb. nov.**
*Cordulegaster coronata* Morton, 1916	*Thecagaster coronata* (Morton, 1916) **comb. nov.**
*Cordulegaster helladica* (Lohmann, 1993)	*Thecagaster helladica* (Lohmann, 1993) **comb. nov.**
*Cordulegaster insignis* Schneider, 1845	*Thecagaster insignis* (Schneider, 1845) **comb. nov.**
*Cordulegaster mzymtae* Bartenev, 1929	*Thecagaster mzymtae* (Bartenev, 1929) **comb. nov.**
*Neallogaster pekinensis* (McLachlan in Selys, 1886)	*Thecagaster pekinensis* (McLachlan in Selys, 1886) **comb. nov.**
*Cordulegaster bilineata* (Carle, 1983)	*Zoraena bilineata* Carle, 1983 **comb. restaur.**
*Cordulegaster diastatops* Selys, (1854)	*Zoraena diastatops* (Selys, 1854) **comb. restaur.**
*Cordulegaster dorsalis* Hagen in Selys, 1853	*Zoraena dorsalis* (Hagen in Selys, 1853) **comb. nov.**
*Cordulegaster erronea* Selys, 1878	*Zoraena erronea* (Selys, 1878) **comb. nov.**
*Cordulegaster maculata* Selys, 1854	*Zoraena maculata* (Selys, 1854) **comb. nov.**
*Cordulegaster obliqua* (Say, 1839)	*Zoraena obliqua* (Say, 1839) **comb. nov.**
*Cordulegaster sarracenia* Abbott & Hibbitz, 2011	*Zoraena sarracenia* (Abbott & Hibbitz, 2011) **comb. nov.**
*Cordulegaster sayi* Selys, 1854	*Zoraena sayi* (Selys, 1854) **comb. nov.**

## Data Availability

The DNA sequences obtained in the course of this study have been submitted to GenBank; for the entry numbers see Table 1.

## Supplementary Materials

The following supporting information can be downloaded at: https://www.mdpi.com/article/10.3390/insects15080622/s1, Supplementary Figure S1. Phylogenetic tree (cladogram) reconstructed the ITS region with the Maximum Likelihood method using IQ-TREE 2.3.5 software. Included are sequences obtained in this study (DNA numbers next to the names) and those retrieved from GenBank (accession numbers next to the names). Supplementary Figure S2. Phylogenetic tree (cladogram) reconstructed the COI region with the Maximum Likelihood method using IQ-TREE 2.3.5 software. Included are sequences obtained in this study (DNA numbers next to the names) and those retrieved from GenBank (accession numbers next to the names). Supplementary Figure S3. The particular gene tree for the ITS region resulted from the simultaneous analysis of the ITS region and COI using the StarBeast3 v 1.1.7 software. Supplementary Figure S4. The particular gene tree for the COI region resulted from the simultaneous analysis of the ITS region and COI using the StarBeast3 v 1.1.7 software. Supplementary Figure S5. The phylogenetic tree reconstructed by the Maximum Likelihood method using IQ-TREE 2.3.5 for the concatenated sequences of ITS region and COI gene fragment. For the sequences obtained in this work, DNA numbers are provided. For those adopted from our previous works [5,34], GenBank accession numbers for the ITS and COI regions are provided next to the names. Supplementary Table S1. Results of species delimitation analysis of representatives of the genus *Anotogaster* as the output of the mPTP program.
