# Supplementary material for: Molecular Phylogeny of the Family Cordulegastridae (Odonata) Worldwide
Source: Insects. 2024 Aug 19;15(8):622. doi: 10.3390/insects15080622 (PMC11354498; doi:10.3390/insects15080622)
Supplement: Supplementary file 1 [file insects-15-00622-s001.zip › Table S1.pdf]

Command: mptp --tree_file Anotogaster_PTP_COI.newick --outgroup Aeshna_grandis_MW490517 --output_file anotogaster_PTP_COI --mcmc 100000000 --multi --mcmc_log --minbr 0.0009330519 --mcmc_sample 10000 --mcmc_runs 4
Number of edges greater than minimum branch length: 145 / 146
Null-model score: 597.999395
Best score for multi coalescent rate: 676.179045
LRT computed p-value: 0.000000
LRT: passed
Number of delimited species: 12

Species 1:
Aeshna_grandis_MW490517

Species 2:
Anotogaster_sieboldii_China_Jiangxi_AB708838
Anotogaster_sieboldii_Japan_main_islands_AB708836
Anotogaster_sieboldii_Japan_main_islands_AB708827
Anotogaster_sieboldii_Japan_main_islands_AB708832
Anotogaster_sieboldii_Japan_main_islands_AB708837
Anotogaster_sieboldii_Japan_main_islands_AB708825
Anotogaster_sieboldii_Japan_main_islands_AB708835
Anotogaster_sieboldii_Japan_main_islands_AB708833
Anotogaster_sieboldii_Japan_main_islands_AB708834
Anotogaster_sieboldii_Japan_main_islands_AB708828
Anotogaster_sieboldii_Japan_main_islands_AB708831
Anotogaster_sieboldii_Japan_KF584973

Species 3:
Anotogaster_chaoi_Vietnam_676
Anotogaster_chaoi_Vietnam_677
Anotogaster_chaoi_Vietnam_AB708802
Anotogaster_sp._Vietnam_AB708845
Anotogaster_chaoi_Vietnam_678

Species 4:
Anotogaster_nipalensis_Nepal_451

Species 5:
Anotogaster_sapaensis_Vietnam_686

Species 6:
Anotogaster_gregoryi_Laos_AB708803
Anotogaster_gregoryi_Thailand_LC366808
Anotogaster_sp._Laos_LC366733

Species 7:
Anotogaster_myosa_China_Shaanxi_671
Anotogaster_sakaii_Vietnam_679
Anotogaster_sakaii_Vietnam_LC366642
Anotogaster_sakaii_Vietnam_680
Anotogaster_sakaii_Vietnam_681
Anotogaster_sp._China_Zhejiang_AB708839

Species 8:
Anotogaster_gigantica_Vietnam_685
Anotogaster_sp._Vietnam_AB708843
Anotogaster_sp._Vietnam_AB708840
Anotogaster_sp._Vietnam_AB708842

Species 9:
Anotogaster_kuchenbeiseri_China_Beijing_AB708822
Anotogaster_kuchenbeiseri_China_Beijing_AB708823

Species 10:
Anotogaster_klossi_China_Guangdong_AB708809
Anotogaster_klossi_China_Guangdong_AB708814
Anotogaster_klossi_China_Zhejiang_AB708808
Anotogaster_klossi_China_Guangdong_AB708810
Anotogaster_klossi_China_Guangdong_AB708812
Anotogaster_klossi_China_Guangdong_AB708816
Anotogaster_klossi_China_Zhejiang_AB708815
Anotogaster_klossi_China_Guangdong_AB708811
Anotogaster_klossi_China_Zhejiang_AB708807
Anotogaster_klossi_Vietnam_AB708841
Anotogaster_klossi_Vietnam_AB708844
Anotogaster_klossi_China_MH064393
Anotogaster_klossi_China_MH064395
Anotogaster_klossi_Laos_AB708820
Anotogaster_klossi_Vietnam_AB708817
Anotogaster_klossi_Vietnam_591
Anotogaster_klossi_Vietnam_683
Anotogaster_klossi_Vietnam_682
Anotogaster_klossi_Japan_Yaeyama_Islands_AB708804
Anotogaster_klossi_Japan_Yaeyama_Islands_AB708813
Anotogaster_klossi_Japan_Yaeyama_Islands_AB708805
Anotogaster_klossi_Japan_Yaeyama_Islands_AB708819
Anotogaster_klossi_Vietnam_AB708818
Anotogaster_klossi_Vietnam_AB708821
Anotogaster_klossi_China_Hunan_592
Anotogaster_klossi_Taiwan_AB708806
Anotogaster_klossi_Taiwan_MN229288
Anotogaster_klossi_Taiwan_MN229281
Anotogaster_klossi_Taiwan_MN229282
Anotogaster_klossi_Taiwan_MN229284
Anotogaster_klossi_Taiwan_MN229285
Anotogaster_klossi_Taiwan_AB711465
Anotogaster_klossi_Taiwan_MN229287
Anotogaster_klossi_Taiwan_MN229283
Anotogaster_klossi_Taiwan_MN229286

Species 11:
Anotogaster_sieboldii_Japan_Amami_Oshima_AB708824
Anotogaster_sieboldii_Japan_Amami_Oshima_AB708829

Species 12:
Anotogaster_sieboldii_Japan_Okinawa_AB708826
Anotogaster_sieboldii_Japan_Okinawa_AB708830
