# Supplementary figures and images for: Molecular Phylogeny of the Family Cordulegastridae (Odonata) Worldwide
Source: Insects. 2024 Aug 19;15(8):622. doi: 10.3390/insects15080622 (PMC11354498; doi:10.3390/insects15080622)

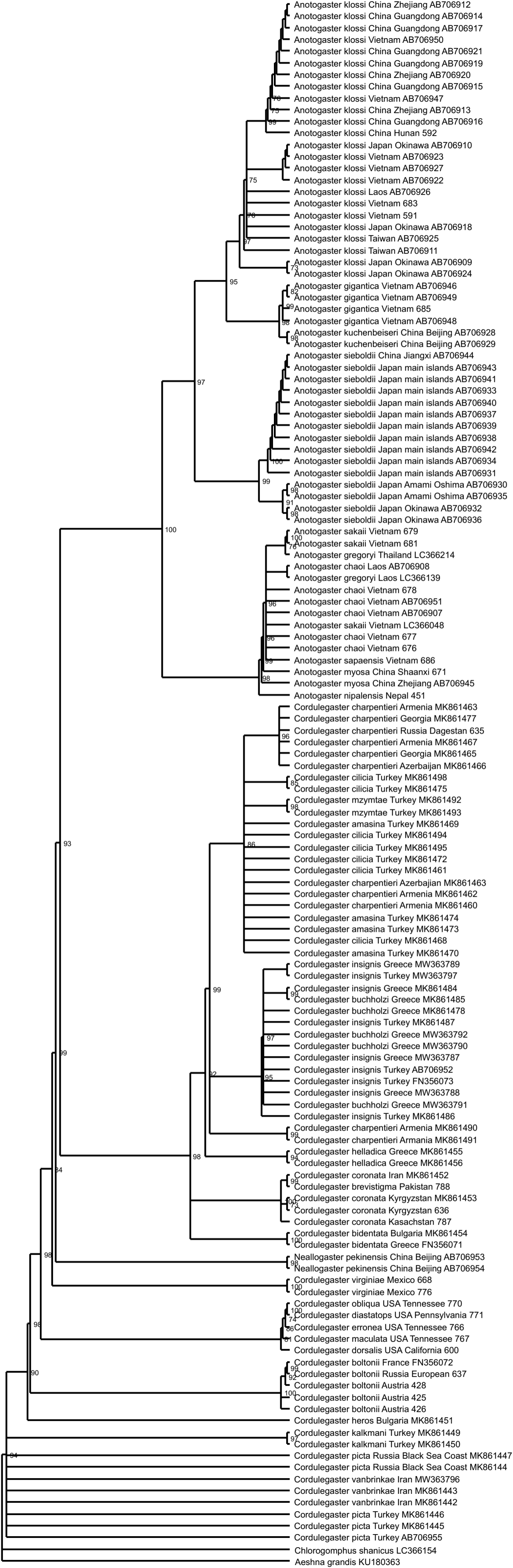

Supplement: Supplementary file 1 [file insects-15-00622-s001.zip › Figure S1.pdf]

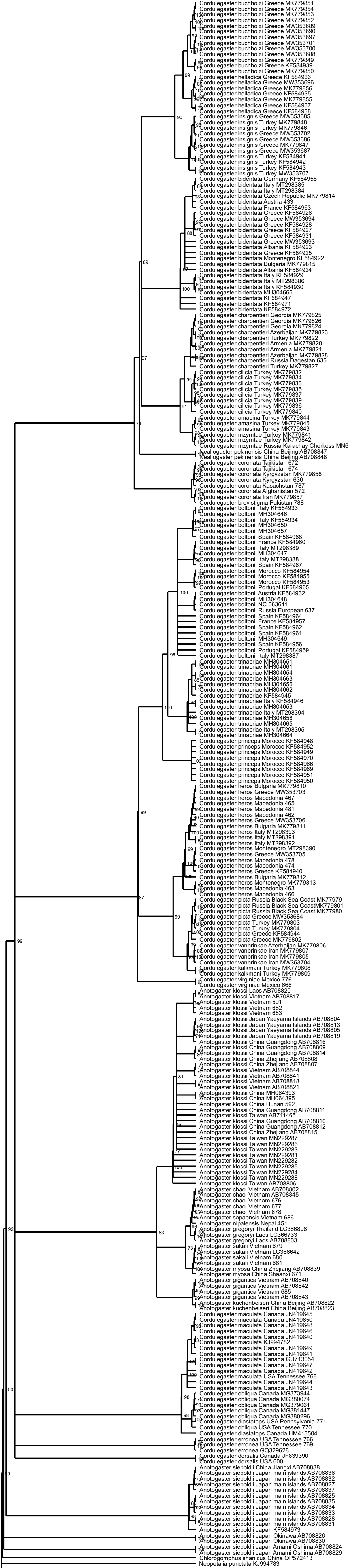

Supplement: Supplementary file 1 [file insects-15-00622-s001.zip › Figure S2.pdf]

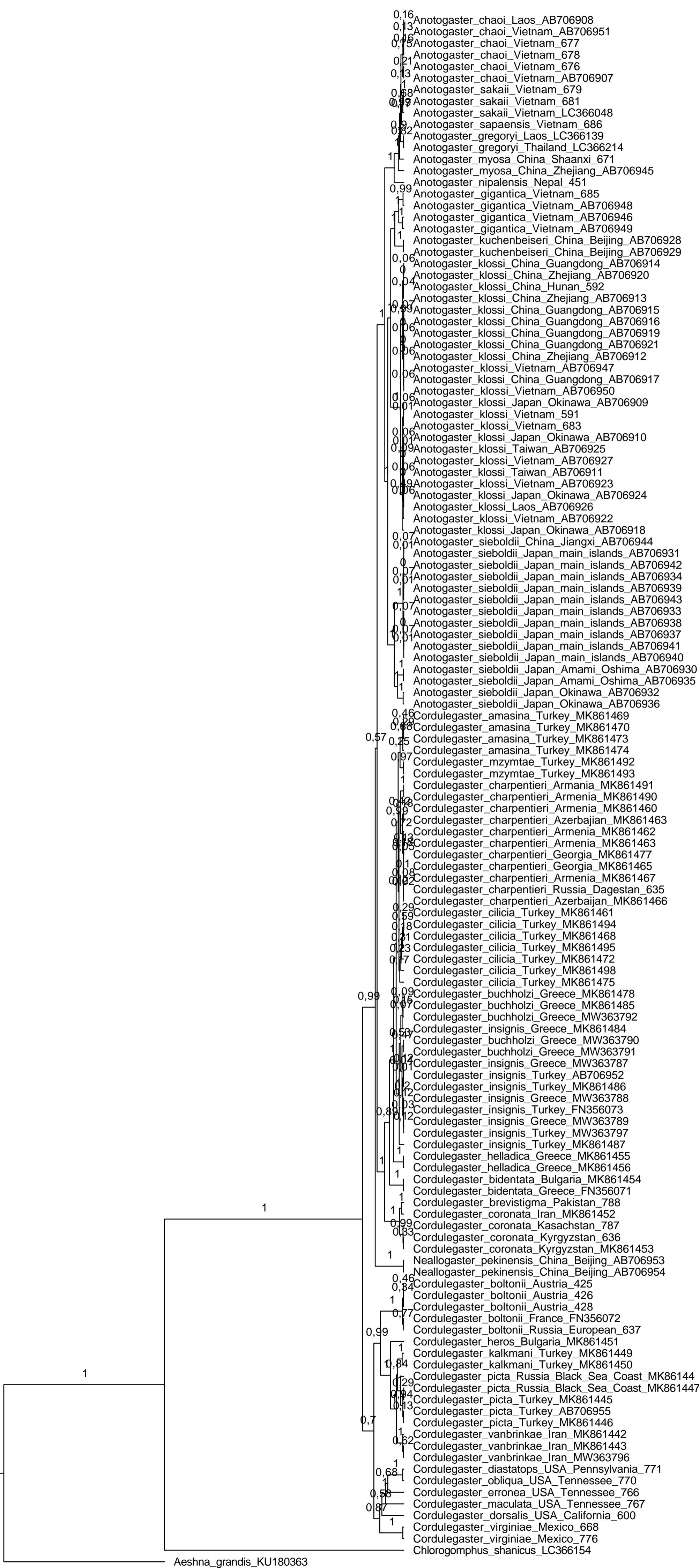

0.03

Supplement: Supplementary file 1 [file insects-15-00622-s001.zip › Figure S3.pdf]

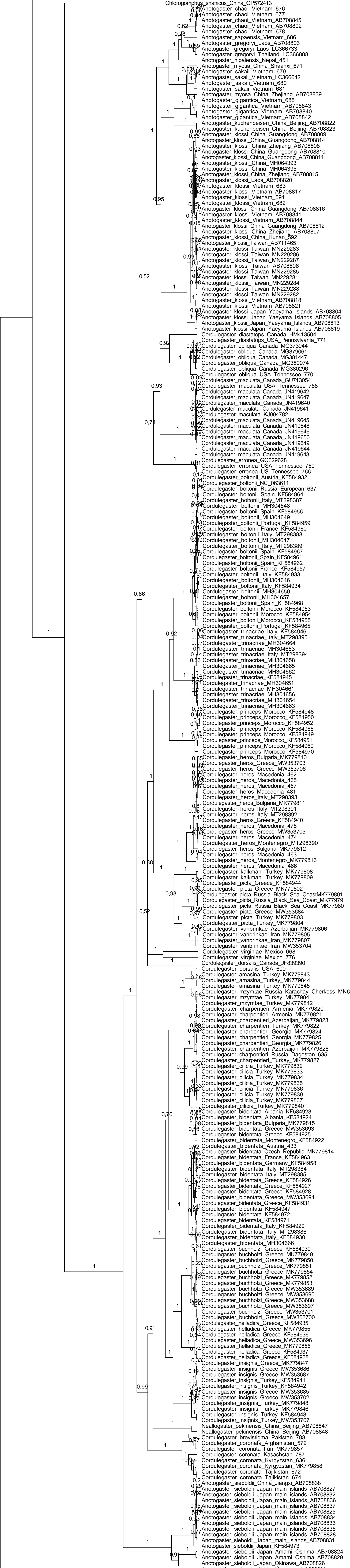

Aeshna\_grandis\_MW490517

Supplement: Supplementary file 1 [file insects-15-00622-s001.zip › Figure S4.pdf]

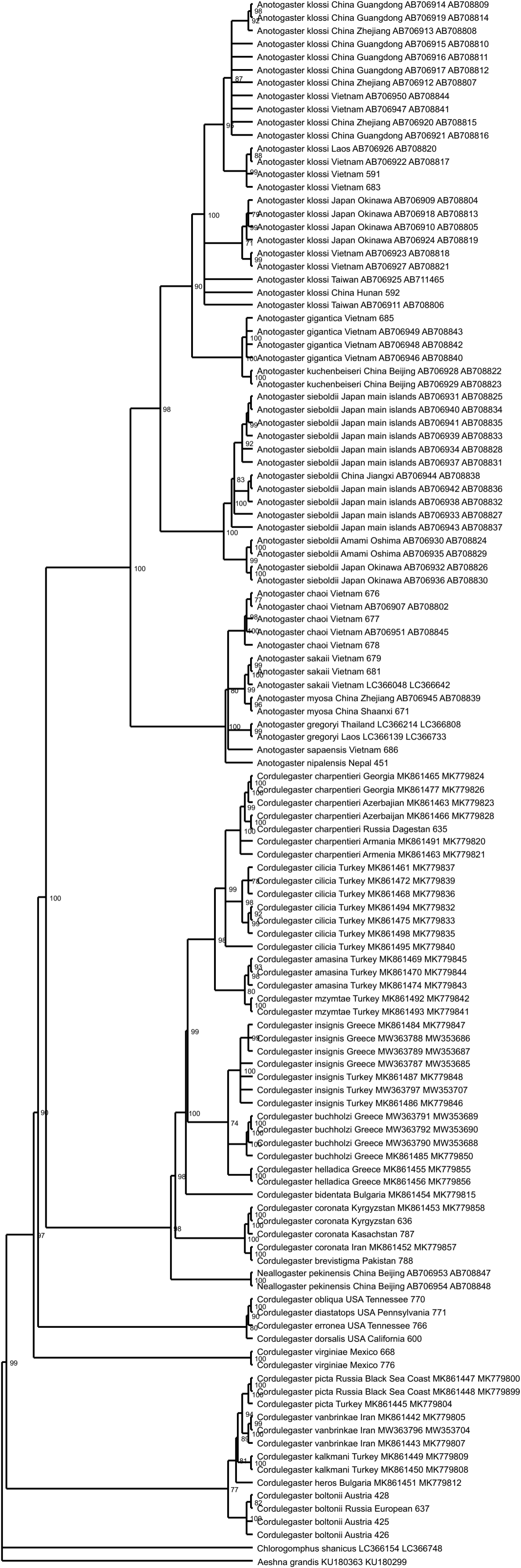

Supplement: Supplementary file 1 [file insects-15-00622-s001.zip › Figure S5.pdf]
